# Biologic therapies for the treatment of large vessel vasculitis: A systematic review and meta-analysis

**DOI:** 10.1371/journal.pone.0314566

**Published:** 2025-03-10

**Authors:** Siyuan Chen, Xiao Cui, Yue Chen, Xiaogang Guo

**Affiliations:** 1 Department of Cardiovascular Diseases, The First Affiliated Hospital, Zhejiang University School of Medicine, Hangzhou, China; 2 Graduate School, Zhejiang University School of Medicine, Hangzhou, China; University of Toledo College of Medicine and Life Sciences, UNITED STATES OF AMERICA

## Abstract

**Objective:**

To summarize the existing evidence from double-blind randomized controlled trials (RCTs) and cohort studies regarding the effects of biologic agents for the treatment of large vessel vasculitis (LVV).

**Methods:**

A systematic review and meta-analysis was conducted using MEDLINE, Embase, Cochrane Central Registry of Controlled Trials, and ClinicalTrials.gov covering the period from database inception to May 3rd, 2023. Double-blind RCTs and cohort studies reporting biologic therapies’ effects on LVV including giant cell arteritis (GCA) and Takayasu’s arteritis (TAK) with outcomes of interest in English were included. The primary outcome of interest was relapse rates during glucocorticoid tapering. The Cochrane Risk of Bias tool 2.0 and the Risk of Bias In Non-randomized Studies of Interventions tool were used for the quality assessment. Random-effects models were used for meta-analysis.

**Results:**

Of the 4599 references retrieved, 10 RCTs regarding GCA, 6 cohort studies, and 2 RCTs regarding TAK were included, comprising 997 participants in total. All the included RCTs were of low risk of bias, while the 6 cohort studies were of moderate to serious risk of bias. Meta-analysis suggested a significant superiority of biologic agents in prolonging relapse-free survival, increasing glucocorticoid taper rate, and decreasing cumulative glucocorticoids dose for both GCA and TAK. Additionally, GCA patients using biologic agents had significantly lower relapse rates and ESR levels with higher remission rates. Trends of favoring biologic agents in reducing relapse rate, ITAS-2010, ITAS-A, ESR, and CRP along with increased remission rate for TAK were also observed.

**Conclusions:**

Biologic agents significantly improved clinical outcomes in LVV by reducing relapse rates, enhancing remission, and enabling safer glucocorticoid tapering, offering an important therapeutic advantage for managing both GCA and TAK. Further well-designed studies and corresponding meta-analyses are needed to validate their long-term efficacy and safety.

## 1. Introduction

Large vessel vasculitis (LVV) is vasculitis with aorta and major branches involved, including two main subtypes, giant cell arteritis (GCA) and Takayasu’s arteritis (TAK) [1]. Chronic inflammation activation could lead to progressive vascular pathological and morphological changes in involved arteries, including stenosis, occlusion, dilation, aneurysm formation, and rupture, resulting in corresponding organ injuries, severe complications, or even death [2,3]. So far, glucocorticoids remain the first line of treatment for GCA and TAK [1]. However, cumulative or large-dose usage of glucocorticoids could inevitably bring adverse effects on patients. Meanwhile, many LVV patients encounter disease relapses during glucocorticoid tapering [4,5]. Given that, therapies that could help decrease glucocorticoids doses for treating LVV patients and keep disease controlled during glucocorticoid tapering are in great request.

Biologic agents are treatments usually recommended for LVV patients with refractory diseases despite adequate usage of conventional treatments [1]. Biologic agents such as tocilizumab, targeting interleukin-6 receptor alpha, have been reported to help LVV patients remain in remission during glucocorticoid tapering [6]. Along with advances in the investigating mechanisms of GCA and TAK, more potential biologic targets have been further identified, and novel biologic agents have been further examined in the LVV population [7–12]. Previously, most studies about the effects of biologic agents for LVV were case reports, and case series, while few of them were well-designed randomized controlled trial or cohort studies, bringing relatively weak evidence for guiding the management of LVV [13]. Considering new evidence appeared in this area recently, we conducted this systematic review and meta-analysis of double-blind randomized controlled trials (RCTs) and cohort studies regarding effects of biologic agents for LVV.

## 2. Methods

The design and conduction of this systematic review and meta-analysis followed the PRISMA statement [14]. The present systematic review and meta-analysis has been registered in PROSPERO (No. 223889).

### 2.1. Eligibility criteria

We included studies that examined the effects of biologic therapies in patients diagnosed with TAK and GCA. Eligible study designs included RCTs and cohort studies that assessed outcomes related to glucocorticoid tapering and disease activity. The primary outcome was relapse rate during glucocorticoid tapering. Secondary outcomes included remission rates, relapse-free survival, cumulative glucocorticoid doses, glucocorticoid tapering dose and rate, and changes in markers of disease activity (e.g., ESR, CRP, ITAS-2010, ITAS-A).

We excluded studies if they (1) did not report results from intention-to-treat analysis in RCTs or (2) did not follow pre-defined standardized glucocorticoid tapering schedules. Only English-language articles were considered, and studies reporting on the same clinical trial were consolidated, using data relevant to the outcomes of interest.

Studies were grouped based on disease type and study design.

### 2.2. Information sources and search strategy

A comprehensive literature search was performed using MEDLINE, Embase, Cochrane Central Register of Controlled Trials (CENTRAL), and ClinicalTrials.gov from inception to May 3, 2023. The search strategies employed population-, intervention-, and study design-related terms (detailed in S1–S3 Tables).

### 2.3. Study selection process

Two investigators independently screened titles and abstracts based on the inclusion and exclusion criteria. Disagreements were resolved through discussion until consensus was reached. Full-text reviews were then conducted for studies meeting the initial screening criteria.

### 2.4. Data extraction

Data extraction was conducted by two independent reviewers using standardized data extraction forms (S1 File). Disagreement was solved by discussion and achieving consensus. For RCTs with more than one intervention and/or control groups with various glucocorticoid tapering protocols, to control the confounding effects of glucocorticoid tapering, only the information of groups with the same glucocorticoid tapering protocol were extracted. For an individual RCT reported the effect of biologics in different dosage or interval time of administration, each arm was analyzed as a separate study. For studies that reported results according to both intention-to-treat and per-protocol analysis, only the results from the intention-to-treat analysis were included. For RCTs with extended open-label single-arm phase after completing the double-blind trial, only the information from the double-blind trial was extracted.

Outcome information was extracted including information reflecting disease activity status during glucocorticoid tapering (relapse rate, remission rate and relapse-free survival), information regarding glucocorticoids doses (cumulative glucocorticoids dose, glucocorticoid tapering dose and glucocorticoid tapering rate), and other information reflecting changes in disease activity status (changes in relevant scores, i.e., ITAS2010 and ITAS-A for TAK, changes in erythrocyte sedimentation rate (ESR) and C-reactive protein (CRP) for both TAK and GCA). Glucocorticoid tapering doses were defined as changes in glucocorticoids between baseline and endpoint. With regards to RCTs in which data was provided at different time points, except for cumulative glucocorticoids dose, data consistent with primary endpoint observation time was obtained. Data for cumulative glucocorticoids of RCTs and all data of cohorts was extracted from the latest measurement. Other extracted variables included characteristics of study populations (age, gender, disease duration at baseline, disease activity status at baseline, prior usage of immunosuppressive agents and other special characteristics), information of intervention and controls (detailed medication, dosage, method of administration and treatment duration), information of concomitant immunosuppressants usage, and follow-up duration. Standardized definitions used in each study including diagnosis criteria used for TAK and GCA, and definitions of remission, relapse and success of achieving glucocorticoid tapering were also extracted.

### 2.5. Risk of bias assessment

The quality of included studies was assessed independently by two reviewers. For RCTs, the Cochrane Risk of Bias (ROB) 2.0 (version 2019) tool was used [15]. For cohort studies, we employed the Risk of Bias In Non-randomized Studies of Interventions (ROBINS-I) tool (version for cohort-type studies 2016) [16]. Discrepancies between reviewers were resolved through discussion.

### 2.6. Data synthesis and meta-analysis

Studies were pooled based on the outcomes of interest, with separate analyses for TAK and GCA. RCTs and cohort studies were analyzed separately as well. For binary outcomes (e.g., relapse rates, remission rates), we used risk ratios (RR) with 95% confidence intervals (CI). For survival data, hazard ratios (HR) were used. If HRs were not reported, they were estimated using methods based on Kaplan-Meier curves [17].

For continuous outcomes, since the cumulative glucocorticoids dose was reported in different units, standardized mean difference (SMD) was used as the effect size statistics to make these studies with different scales comparable, while for glucocorticoid tapering dose, and changes in ESR, CRP, ITAS-2010 and ITAS-A, weighted mean difference (WMD) were used. For data reported in median, range and/or interquartile range, Luo’s method and Wan’s method were used for estimating the sample mean and standard deviation respectively as recommended by the Cochrane Handbook for Systematic Reviews of Interventions (version 6.1, 2020) [18–21]. For SD change not reported in studies, an estimation of the SD change was calculated using the following formula: SD =  square root [(SD baseline) 2 +  (SD final) 2 − (2 R ×  SD baseline ×  SD final)], and a correlation coefficient (R) =  0.5 according to the guidelines of the Cochrane Collaboration [22]. Considering clinical heterogeneity across studies, random-effects models were used for meta-analysis of outcomes of interest. Forest plots were used to visually display the results of individual studies and syntheses. Statistical heterogeneity across studies was reported with heterogeneity chi-squared value, p-value, and I^2^. Publication bias was assessed using funnel plots and the Egger test. All statistical analyses were conducted using Stata version 15.1.

## 3. Results

### 3.1. Database search and selection results

**Fig 1:**
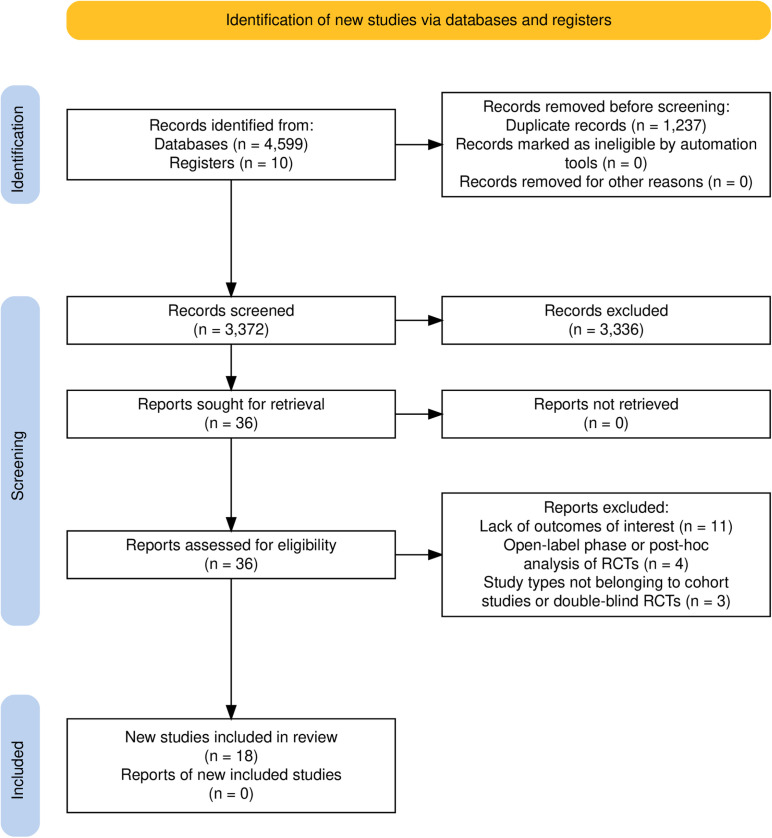
Flowchart of the database search and selection results. RCTs =  randomized controlled trials.

Database searches using MEDLINE, Embase, and CENTRAL identified 4599 references. Database searches using ClinicalTrials.gov identified 10 items for a supplement. 3372 references remained after excluding repetitions. 3336 references not within the scope of this review were excluded after reading study titles and/or abstracts. After reviewing full articles of the remaining 36 references, 11 studies were excluded due to lack of outcomes of interest, 4 due to the open-label phase or post-hoc analysis of RCTs and 3 due to study types not belonging to cohort studies or double-blind RCTs [23–40]. 18 studies were finally included, including 10 RCTs regarding GCA, 6 cohort studies, and 2 RCTs regarding TAK [6,41–57]. No cohort study of GCA met the inclusion criteria of this review (Fig 1) [58].

### 3.2. Characteristics and quality assessment of included studies

The characteristics and quality assessment of included studies regarding LVV are shown in Table 1 [ 6,41–57]. Details in prior and concomitant immunosuppressant usage, treatment administration dose and duration, and disease activity status of the study population were listed in S4 Table.

**Table 1:** Characteristics and quality assessment of included studies regarding effects of biologic agents for the treatment of large vessel vasculitis.

Study	Design	Drug	Comparison	Number of Study population		Age (years), mean ± S.D.		Gender, female, n (%)		Follow-up duration	Risk of Bias^**^
				Biologics	Comparator	Biologics	Comparator	Biologics	Comparator		
Hoffman, G. S. et al. 2007	RCT	IFX	Placebo	28	16	70.9 ± 67.42	70.59 ± 9.75	24 (86)	11 (69)	22 weeks	low
Martínez-Taboada, V. M. et al. 2007	RCT	ETA	Placebo	8	9	74.5 ± 5.7	74.4 ± 6.8	6 (75)	8 (88.9)	15 months	low
Seror, R. et al. 2013	RCT	ADA	Placebo	34	36	74.5 (69–78)^*^	74.5 (67–80.5)^*^	24 (70.6)	28 (77.8)	52 weeks	low
Villiger, P. M. et al. 2016	RCT	TCZ	Placebo	20	10	71.3 ± 8.9	68.8 ± 16.9	13 (65)	8 (80)	52 weeks	low
Langford, C. A. et al. 2017	RCT	ABA	Placebo	20	21	65.05 ± 6.10	71.20 ± 8.55	16 (80)	21 (100)	12 months + 24 weeks	low
Stone, J. H. et al. 2017	RCT	TCZ	Placebo	TCZ Q1W group, 100; TCZ Q2W group, 50	50	TCZ Q1W group, 69.5 ± 8.5; TCZ Q2W group, 69.4 ± 8.2	69.3 ± 8.1	TCZ Q1W group, 78 (78); TCZ Q2W group, 35 (70)	38 (76)	52 weeks	low
Schmidt, W. A. et al. 2020	RCT	SRK	Placebo	SRK 100mg Q2W, 42; SRK 50mg Q4W, 26	27	SRK 100mg Q2W, 70.5 ± 7.3; SRK 50mg Q4W, 67.5 ± 9.5	71.6 (7.1)	SRK 100mg Q2W, 31 (73.8); SRK 50mg Q4W, 19 (73.1)	23 (85.2)	52 weeks	low
Cid, M. C. et al. 2022	RCT	Mavrilimumab	Placebo	42	28	69.7 ± 7.0	69.7 ± 8.3	32 (76)	18 (64)	26 weeks + 12 weeks	low
NCT03600805 2022	RCT	Sarilumab	Placebo	Sarilumab 150mg Q2W, 14; Sarilumab 200mg Q2W, 27	14	Sarilumab 150mg Q2W, 67.1 ± 7.9; Sarilumab 200mg Q2W, 73.4 ± 8.6	69.5 ± 5.4	Sarilumab 150mg Q2W, 13(92.9); Sarilumab 200mg Q2W, 23(85.2)	9(64.3)	52 weeks	low
NCT03765788 2023	RCT	SCK	Placebo	27	25	76.4 ± 5.31	69.6 ± 8.02	17(63)	18(72)	52 weeks	low
Langford, C. A. et al. 2017	RCT	ABA	Placebo	11	15	33.67 ± 12.56	31.92 ± 10.78	9 (82)	13 (87)	24 weeks after termination	low
Nakaoka, Y. et al. 2018	RCT	TCZ	Placebo	18	18	31.1 ± 18.1	30.8 ± 13.1	16 (88.9)	15 (83.3)	median duration, 19 weeks in the TCZ group, 12.87 weeks in the placebo group	low
Kong, X. et al. 2018	prospective cohort	TCZ	CTX	9	15	32.11 ± 11.76	43.00 ± 16.68	10 (66.67)	8 (88.89)	6 months	serious
Pan, L. et al. 2020	retrospective cohort	TCZ	CTX, MTX	11	11	35.64 ± 10.57	38.40 ± 15.32	10 (90.9)	11 (100.0)	6 months	serious
Kong, X. et al. 2022	prospective cohort	TOF	MTX	27	26	31.11 ± 9.58	33.50 ± 14.89	22(81.5)	23(88.5)	12 months	moderate
Liao, H. et al. 2022	retrospective cohort	TCZ	CTX	31	32	35.2 ± 12.5	40.8 ± 10.1	29(93.5)	28(87.5)	6 months	serious
Wang, J. et al. 2022	prospective cohort	TOF	LEF	32	35	30.94 ± 9.03	33.23 ± 12.07	26(81.25)	30(85.71)	12 months	serious
Yoshida, S. et al. 2023	retrospective cohort	TCZ	MTX(33.3%); AZA (27.8%)	14	18	28.87 ± 26.36	38.63 ± 47.46	12(85.7)	15(83.3)	median duration, 40 weeks in the TCZ group, 37 weeks in the non-TCZ group	serious

* expressed as median (95% CI).

** ROB 2.0 for RCT, ROBINS-I for cohort studies. ABA =  abatacept; ADA =  adalimumab; AZA =  azathioprine; CTX =  cyclophosphamide; ETA =  etanercept; GCA =  giant cell arteritis; IFX =  infliximab; LEF =  leflunomide; MTX =  methotrexate; NR =  not reported; RCT =  randomized controlled trials; SCK =  secukinumab; SRK =  sirukumab; TAK =  Takayasu’s arteritis; TCZ =  tocilizumab; TOF =  tofacitinib.

All the included RCTs, 10 for GCA and 2 for TAK were of low risks of bias according to the quality assessment using ROB 2.0 tool, suggesting quite strong strength of evidence. However, the 6 included cohort studies of TAK were of moderate to serious risks of bias according to the quality assessment using the ROBINS-I tool, suggesting concerns should be paid when illustrating those results.

### 3.3. Effects of biologic agents for GCA on relapse rate, remission rate and relapse-free survival in RCTs

**Fig 2:**
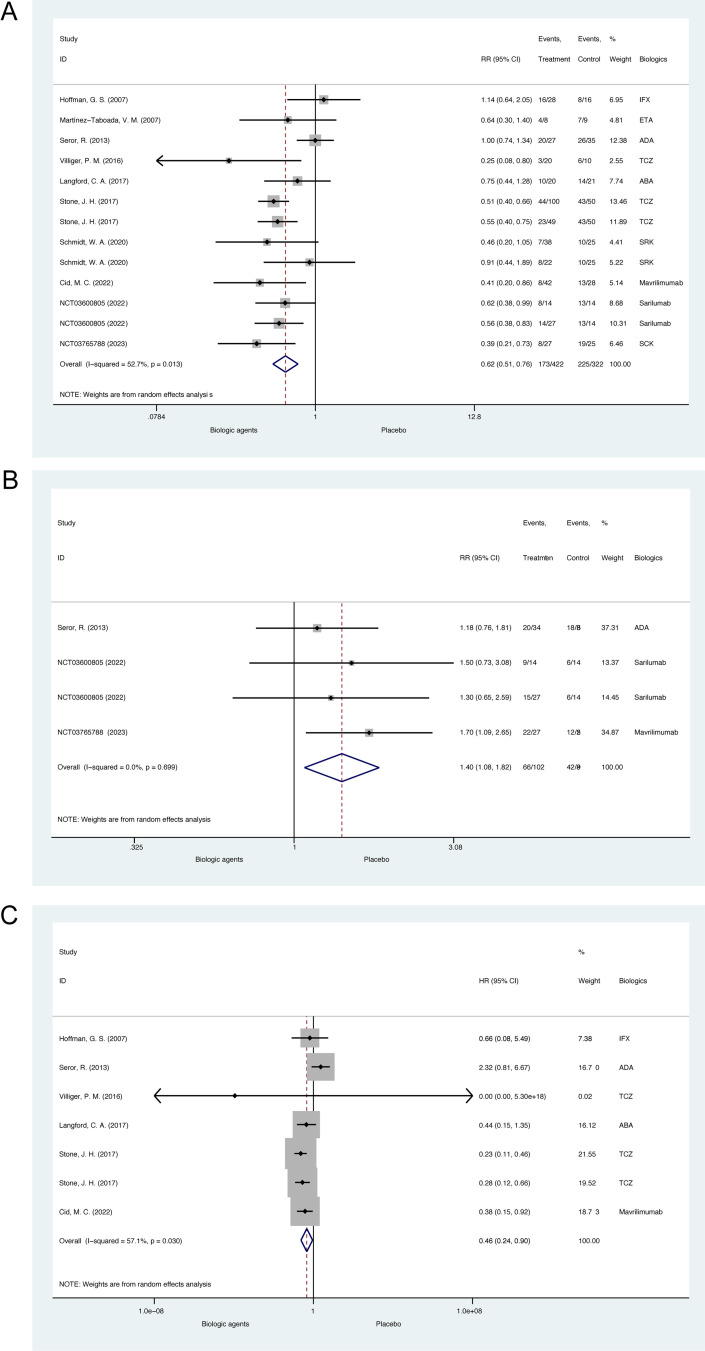
Meta-analysis of biologic agents on disease activity status during glucocorticoid tapering for giant cell arteritis in double-blind randomized controlled trials. (A) The forest plot of relapse rate. (B) The forest plot of remission rate. (C) The forest plot of relapse-free survival. ABA =  abatacept; ADA =  adalimumab; ETA =  etanercept; IFX =  infliximab; SCK =  secukinumab; SRK =  sirukumab; TCZ =  tocilizumab.

All 10 RCTs (N =  674) regarding GCA with 13 pairs of comparison reported the effects of biologic agents on relapse rate during glucocorticoid tapering, which were all of low risk of bias [6,41–46,55–57]. According to the preset protocol which has been demonstrated in the Methods section, data of more than one biologic agent group with different dosages or interval time of administration were presented and included in the meta-analysis in studies including Stone, J. H. et al. 2017, Schmidt, W. A. et al. 2020 and NCT03600805 2022 [6,43,57]. The pooled relative risk for relapse of GCA patients treated with biologics compared to placebo was 0.62 (95% CI, 0.51 to 0.76, p <  0.001; heterogeneity chi-squared =  25.37, p =  0.013, I^2^ =  52.7%, Fig 2A), suggesting biologics could significantly decrease relapse rates for GCA during glucocorticoid tapering. The funnel plot and Egger’s test for small-study effects suggested no significant publication bias (p =  0.654, S1 Fig).

3 RCTs (N =  177) with 4 comparison pairs reported remission rate [42,56,57]. The pooled relative risk for remission of GCA patients treated with biologics compared to placebo was 1.40 (95% CI, 1.08 to 1.82, p =  0.012; heterogeneity chi-squared =  1.43, p =  0.699, I^2^ =  0%, Fig 2B), also suggesting biologics could significantly increase remission rates for GCA. The funnel plot and Egger’s test suggested no significant publication bias, either (p =  0.963, S2 Fig).

For time-to-event analysis, 6 RCTs (N =  411) with 7 comparison pairs reported hazard ratio (HR), or instead at least presented Kaplan-Meier curves of relapse-free survival during glucocorticoid tapering [6,41,42,45,46,55]. The pooled HR for relapse-free survival of GCA patients treated with biologics compared to placebo was 0.46 (95% CI, 0.24 to 0.90, p =  0.024; heterogeneity chi-squared =  13.99, p =  0.030, I^2^ =  57.1%, Fig 2C), suggesting biologics could significantly prolong relapse-free survival for GCA during glucocorticoid tapering. The funnel plot and Egger’s test suggested no significant publication bias, either (p =  0.663, S3 Fig).

### 3.4. Effects of biologic agents for GCA on glucocorticoid tapering in RCTs

**Fig 3:**
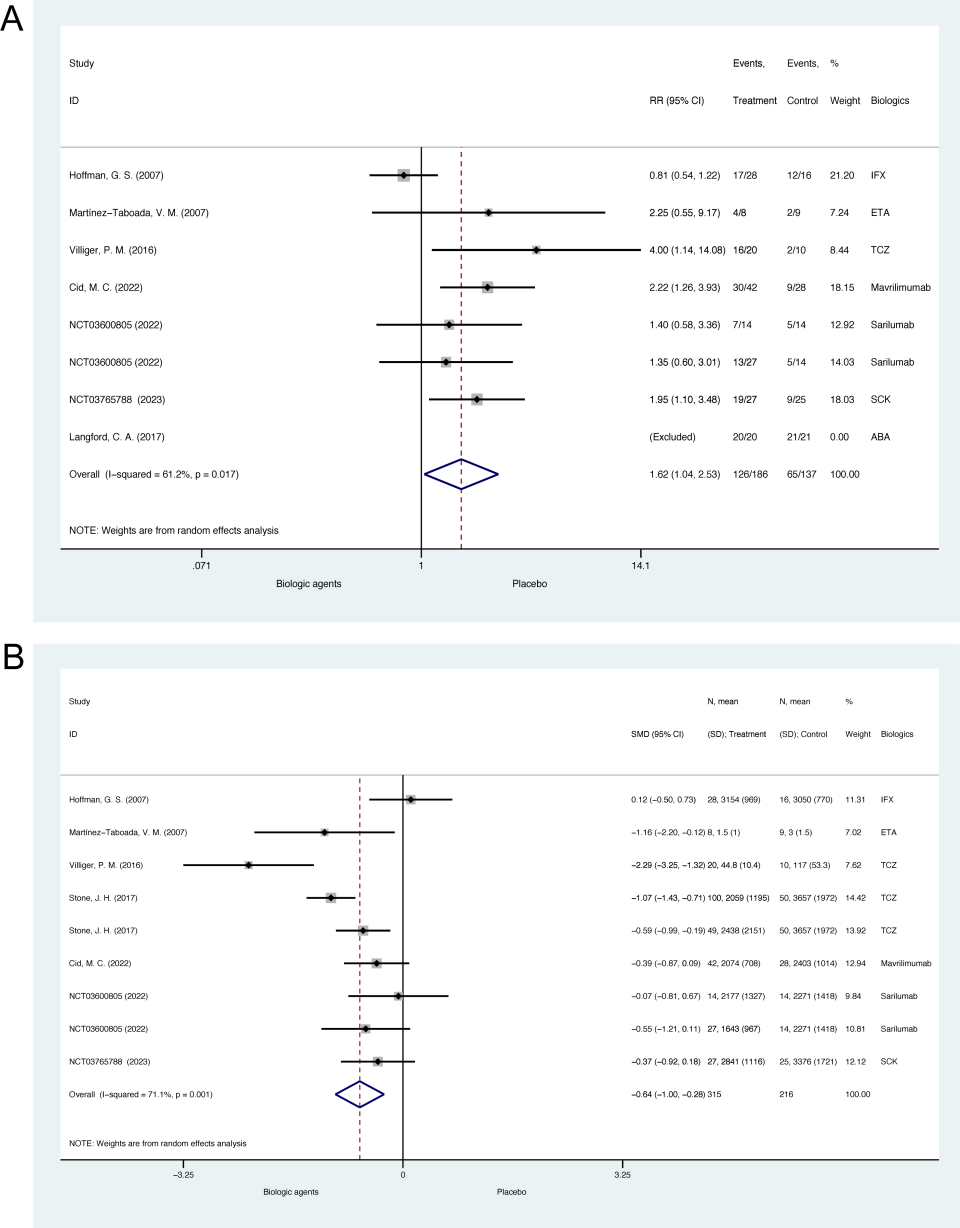
Meta-analysis of biologic agents on glucocorticoid tapering for giant cell arteritis in double-blind randomized controlled trials. (A) The forest plot of glucocorticoid taper rate. (B) The forest plot of cumulative glucocorticoid dose. ABA =  abatacept; ETA =  etanercept; IFX =  infliximab; SCK = secukinumab; TCZ =  tocilizumab.

7 RCTs (N =  294) with 8 comparison pairs reported the effects of biologic agents on glucocorticoid tapering rate for GCA [41,44–46,55–57]. In Langford, C. A. et al. 2017, all patients had discontinued prednisone at week 28 according to a predefined standardized prednisone tapering protocol. This study was thus excluded from the meta-analysis [45]. For remained studies, the pooled relative risk for glucocorticoid tapering rate was 1.62 (95% CI, 1.04 to 2.53, p =  0.034; heterogeneity chi-squared =  15.46, p =  0.017, I^2^ =  61.2%, Fig 3A), suggesting a significant higher rate of successful glucocorticoid tapering. The funnel plot and Egger’s test for small-study effects suggested no significant publication bias (p =  0.134, S4 Fig). It should be noted that the success of achieving glucocorticoid tapering was defined differently in these studies (S5 Table).

As to glucocorticoid taper dosage, only Seror, R. et al. 2013 reported the results with the between-group differences were -0.01mg/kg per day (95% CI -0.07 to 0.05, p =  0.77) over the week 26 period and -0.02mg/kg per day (95% CI -0.10 to 0.07, p =  0.71) over the week 52, indicating no significant difference between biological group and placebo group [42].

7 RCTs (N =  468) with 9 comparison pairs reported the effects of biologic agents on cumulative glucocorticoid dose for GCA [6,41,44,46,55–57]. The pooled result of changes in cumulative glucocorticoids dose is -0.64 (95%CI, -1.00 to -0.28, p <  0.001; heterogeneity chi-squared =  27.68, p =  0.001, I^2^ =  71.1%, Fig 3B), suggesting biologics could significantly reduce cumulative glucocorticoids dose for GCA patients. According to the funnel plot and Egger’s test, there was no significant publication bias (p =  0.907, S5 Fig).

### 3.5. Effects of biologic agents for GCA on ESR and CRP in RCTs

2 RCTs with 3 comparison pairs (N =  107) regarding GCA reported the effects of biologic agents on changes in ESR and CRP from baseline to the follow-up [56,57]. The pooled result for ESR is -20.67 (95% CI, -31.29 to -10.06, p <  0.001; heterogeneity chi-squared =  3.81, p =  0.149, I^2^ =  47.5%, S6A Fig), suggesting biologics could significantly reduce ESR levels in GCA patients. The pooled result for CRP is -0.93 (95%CI, -6.31 to 4.46, p <  0.737; heterogeneity chi-squared = 0.04, p =  0.982, I^2^ =  0.0%, S7A Fig), suggesting a mild while non-significant trend towards favoring biologics. According to the Funnel plot and Egger test, there was no significant publication bias, either (ESR, p =  0.780, S6B and S6C Fig; CRP, p =  0.373, S7B and S7C Fig).

### 3.6. Effects of biologic agents for TAK on relapse rate, relapse-free survival, and glucocorticoid tapering in RCTs

Both of the two RCTs (N =  62) regarding TAK reported the effects of biologic agents on relapse rate [47,48]. In Langford, C. A. et.al 2017 studying the abatacept compared to placebo in TAK patients, the relative risk for relapse was 1.091 (95% CI, 0.656 to 1.815) [48]. In Nakaoka, Y. et al. 2018 studying tocilizumab compared to placebo, the relative risk for relapse was 0.727 (95% CI, 0.386 to 1.372) [47]. The pooled relative risk for relapse of TAK patients treated with biologics compared to placebo was 0.93 (95% CI, 0.62 to 1.39, p =  0.723; heterogeneity chi-squared =  1.02, p =  0.314, I^2^ =  1.5%, S8A Fig), suggesting a mild while non-significant trend towards favoring biologics. The funnel plot suggested no significant publication bias (S8B and S8C Fig).

For time-to-event analysis, Nakaoka, Y. et al. 2018 reported HR for relapse, while Langford, C. A. et.al 2017 provided Kaplan-Meier curves of relapse-free survival during glucocorticoid tapering [47,48]. The pooled hazard ratio for relapse-free survival of TAK patients treated with biologics compared to placebo was 0.41 (95% CI, 0.15 to 1.09, p =  0.074; heterogeneity chi-squared =  0.00, p =  0.972, I^2^ =  0.0%, S9A Fig), suggesting a mild while non-significant trend towards favoring biologics in prolonging relapse-free survival. The funnel plot suggested no significant publication bias (S9B and S9C Fig).

Nakaoka, Y. et al. 2018 (N =  36) reported the effects of biologic agents on successful glucocorticoid tapering for TAK. 8 (44.4%) of 18 subjects treated with tocilizumab successfully decreased their glucocorticoid dose to the minimum dose, while only 3 (16.67%) subjects achieve that in the control arm [47].

### 3.7. Effects of biologic agents for TAK on relapse rate, remission rate, and relapse-free survival in cohort studies

**Fig 4:**
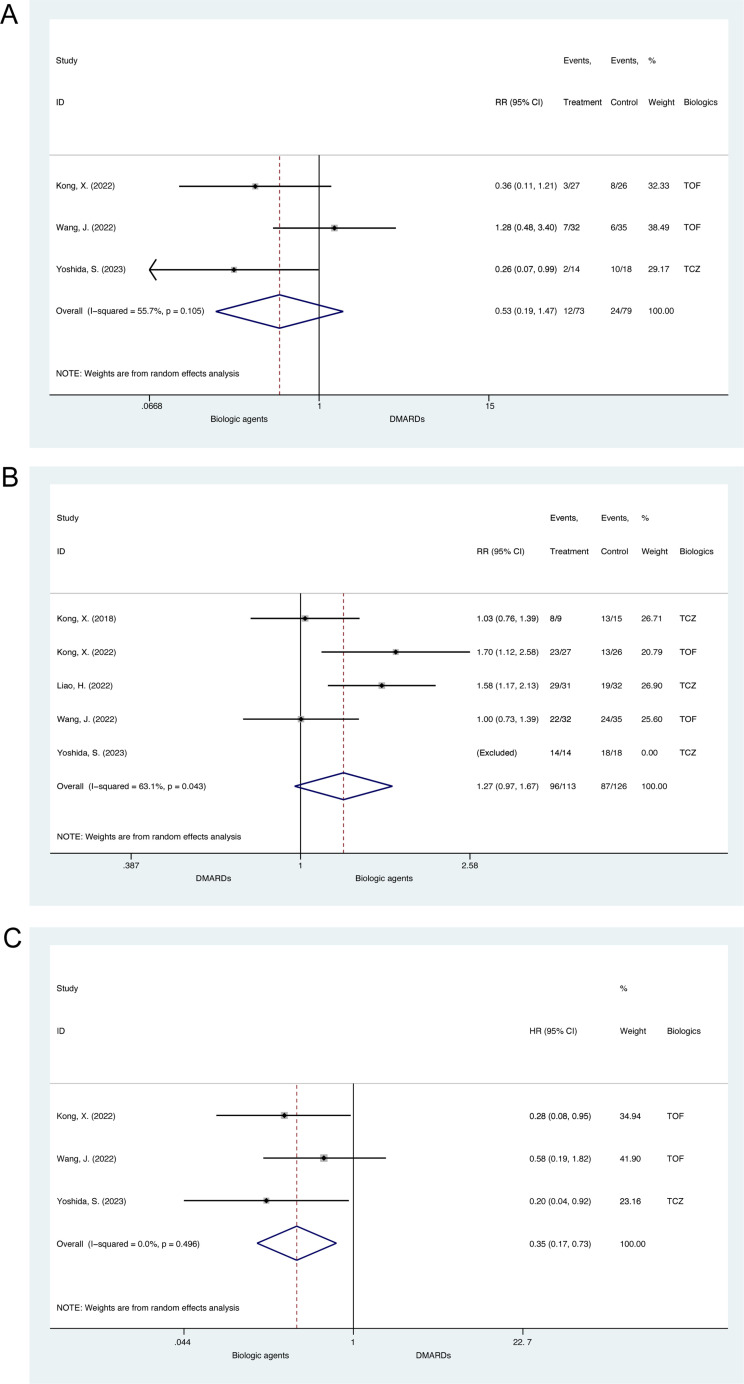
Meta-analysis of biologic agents on disease activity status during glucocorticoid tapering for Takayasu’s arteritis in cohort studies. (A) The forest plot of relapse rate. (B) The forest plot of remission rate. (C) The forest plot of relapse-free survival. TCZ =  tocilizumab; TOF =  tofacitinib.

6 cohort studies (N =  261) regarding TAK that were finally included in this review [49–54]. 3 cohorts reported the effects of biologic agents on relapse rate during glucocorticoid tapering, which were of moderate to serious risk of bias [51,52,54]. The pooled relative risk for relapse of TAK patients treated with biologic agents compared to disease-modifying antirheumatic drugs (DMARDs) was 0.53 (95% CI, 0.19 to 1.47, p =  0.223; heterogeneity chi-squared =  4.51, p =  0.105, I^2^ =  55.7%, Fig 4A), suggesting a mild while non-significant trend towards favoring biologics. The funnel plot and Egger’s test for small-study effects suggested no significant publication bias (p =  0.096, S10 Fig). It should be noted that definitions of relapse were different across studies (S6 Table).

5 cohorts (N =  239) reported remission rates, which were of moderate to serious risk of bias [50–54]. In Yoshida, S. et al. 2023, all patients achieved remission and thus the study was excluded from the meta-analysis [51]. The pooled relative risk for remission of TAK patients treated with biologics compared to DMARDs was 1.27 (95% CI, 0.97 to 1.67, p =  0.085; heterogeneity chi-squared =  8.14, p =  0.043, I^2^ =  63.1%, Fig 4B), suggesting a mild while non-significant trend towards favoring biologics. The funnel plot and Egger’s test for small-study effects suggested no significant publication bias (p =  0.572, S11 Fig). It should be noted that definitions of remission were different across studies (S7 Table).

For time-to-event analysis, 3 cohort studies (N =  152) regarding TAK reported HR for relapse-free survival during glucocorticoid tapering, which were of moderate to serious risk of bias [51,52,54]. The pooled hazard ratio for relapse-free survival of TAK patients treated with biologics compared to DMARDs was 0.35 (95% CI, 0.17 to 0.73, p =  0.005; heterogeneity chi-squared =  1.40, p =  0.496, I^2^ =  0.0%, Fig 4C), suggesting biologics could significantly prolong relapse-free survival for TAK patients. The funnel plot and Egger’s test for small-study effects suggested no significant publication bias (p =  0.366, S12 Fig).

### 3.8. Effects of biologic agents for TAK on glucocorticoid tapering in cohort studies

**Fig 5:**
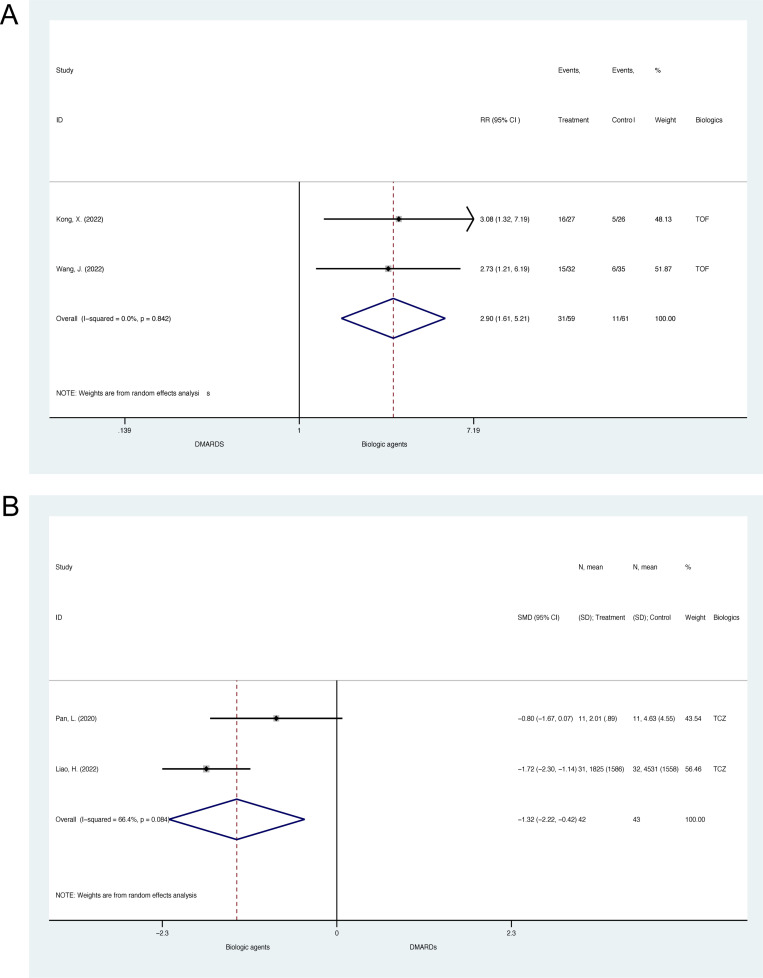
Meta-analysis of biologic agents on glucocorticoid tapering for Takayasu’s arteritis in cohort studies. (A) The forest plot of glucocorticoid taper rate. (B) The forest plot of cumulative glucocorticoid dose. TCZ =  tocilizumab; TOF =  tofacitinib.

2 cohort studies (N =  120) reported the effects of biologic agents on glucocorticoid tapering rate for TAK, which were of moderate to serious risk of bias [52,54]. The pooled relative risk for patients with a daily dose ≤  7.5mg at 12 months was 2.90 (95% CI, 1.61 to 5.21, p <  0.001; heterogeneity chi-squared =  0.04, p =  0.842, I^2^ =  0.0%, Fig 5A), suggesting a significant trend towards favoring biologics. The funnel plot suggested no significant publication bias (S13 Fig). Wang, J. et al. 2022 also reported the taper rate of less than 5mg at 12 months, which is 11 (34.38%) for the tofacitinib group and 0 (0.00%) for the leflunomide group (p <  0.01) [52]. However, Yoshida, S. et al. 2023 found no significant difference in terms of rates of patients who achieved prednisolone less than 10mg per day (p =  0.425) [51].

2 cohort studies (N =  85) reported the effects of biologic agents on cumulative glucocorticoids dose for TAK, which were all of serious risk of bias [49,53]. The pooled SMD for the result of cumulative glucocorticoids dose is -1.32 (95% CI, -2.22 to -0.42, p =  0.004; heterogeneity chi-squared =  2.98, p =  0.084, I^2^ =  66.4%, Fig 5B), suggesting biologics could reduce cumulative glucocorticoids dose significantly. According to the funnel plot, there was no significant publication bias (S14 Fig).

4 cohort studies (N =  162) reported the effects of biologic agents on changes in glucocorticoid dose for TAK, which were of moderate to serious risk of bias [49,50,53,54]. The pooled WMD for changes in glucocorticoids dose was 6.21 (95% CI, 0.76 to 11.66, p =  0.026; heterogeneity chi-squared =  3.12, p =  0.373, I^2^ =  3.9%, S15A Fig), favoring DMARDs. Given that all the treatment groups of these 4 cohorts showed lower glucocorticoid doses at baseline compared to disease-modifying antirheumatic drugs (DMARDs), the result may be confounded by the unbalanced baseline status. The funnel plot and Egger’s test for small-study effects suggested no significant publication bias (p =  0.650, S15B and S15C Fig). Wang, J. et al. 2022 and Yoshida, S. et al. 2023 both reported that no significant difference was found in glucocorticoids reduction between the biologics group and the DMARDs group at each time point [51,52].

### 3.9. Effects of biologic agents for TAK on activity index, ESR, and CRP in cohort studies

ITAS-2010, ITAS-A, CRP, and ESR were well recognized as indicators for disease activity status of TAK. 3 cohort studies (N =  109) regarded TAK reported the changes in ITAS-2010, which were all of serious risk of bias [49,50,53]. The pooled WMD for changes in ITAS-2010 was -0.67 (95% CI, -3.52 to 2.19, p =  0.647; heterogeneity chi-squared =  5.72, p =  0.057, I^2^ =  65.0%, S16A Fig), suggesting a mild while non-significant trend towards favoring biologics. The funnel plot and Egger’s test for small-study effects suggested no significant publication bias (p =  0.079, S16B and S16C Fig). These 3 cohorts also reported changes in ESR and CRP [49,50,53]. The pooled WMD was -15.71 (95% CI, -36.82 to 5.40 p =  0.145; heterogeneity chi-squared =  8.26, p =  0.016, I^2^ =  75.8, S17A Fig) and -1.96 (95% CI, -10.13 to 6.21 p =  0.638; heterogeneity chi-squared =  3.40, p =  0.183, I^2^ =  41.2%, S18A Fig) respectively, in consistent with that of ITAS-2010, also suggesting a mild while non-significant trend towards favoring biologics. The funnel plot and Egger’s test for small-study effects suggested no significant publication bias, either (ESR: p =  0.381, S17B and S17C Fig; CRP: p =  0.196, S18B and S18C Fig). Moreover, two studies showed figures and p values of changes in ESR and CRP without providing detailed data [50,52]. Kong, X. et al. 2018 observed a significant reduction of ESR and CRP in the biologics group while not in the DMARDs group [50]. However, Wang, J. et al. 2022 reported no differences in the ESR or CRP level after treatment [52].

2 cohort studies (N =  85) regarded TAK reported the changes in ITAS-A, which were all of serious risk of bias [49,53]. The pooled WMD for changes in ITAS-A was -0.40 (95%CI, -3.64 to 2.83 p =  0.807; heterogeneity chi-squared =  1.78 p =  0.182, I^2^ =  43.9%, S19A Fig), suggesting a mild while non-significant trend towards favoring biologics as well. The funnel plot suggested no significant publication bias, either (S19B and S19C Fig).

## 4. Discussion

In this systematic review and meta-analysis, we included well-designed double-blind RCTs and cohort studies regarding the effects of biologic agents for LVV, and found that biologic agents could significantly prolonging relapse-free survival, increasing glucocorticoid taper rate, and decreasing cumulative glucocorticoid dose for both GCA and TAK. The quality assessment suggested a low risk of bias for all included double-blind RCTs and moderate to serious risk of bias for the cohort studies, thus bring relatively strong evidence for reviewing the effects of biologic agents for LVV.

Glucocorticoids are the cornerstone for the treatment of GCA and TAK. However, the adverse events during long-term use of glucocorticoids and undesired disease relapses during glucocorticoid tapering are unavoidable issues [4,5]. Evidence regarding biologic agents’ benefits for disease activity control during glucocorticoid tapering in LVV patients has been continuously accumulated [6,41,43–45,51,54]. In recent years, novel biological agents and studies focusing on their effects on LVV have emerged [59,60]. In the present studies, we not only include classic biological agents such as infliximab and tocilizumab, but also novel ones like mavrilimumab, bringing comprehensive review and corresponding analysis.

Our study demonstrates that biologic therapies could significantly alter the management of LVV. By effectively targeting underlying inflammatory pathways, these therapies could lead to a more tailored treatment approach, minimizing glucocorticoid dosages while maintaining effective disease control. This shift not only improves patient outcomes but also enhances their quality of life by reducing the burden of glucocorticoid-related complications.

Even though GCA and TAK share quite a lot of similarities including clinical, histopathological, and imaging features, they are widely recognized as two different clinical entities [9,61]. Our meta-analysis and review found that compared with TAK, GCA can benefit more from the biologic therapies, which is possibly explained by different pathogenesis and mechanisms between these two LVV. GCA is typically characterized by granulomatous lesions mediated by CD4 + T cells and macrophages, along with specific cytokine profiles, which make it more amenable to targeted biologic interventions. For example, tocilizumab, an IL-6 receptor antagonist, has been shown to reduce relapse rates and lower the glucocorticoid doses required for disease control [6]. Similarly, mavrilimumab, which blocks the GM-CSF receptor-α, can prolong glucocorticoid-induced remission and reduce relapse rates [55]. These findings underscore the significant roles of the IL-6 and GM-CSF receptor signaling pathways in sustaining inflammatory activity. In contrast, TAK exhibits a more complex and variable pathophysiology, characterized not only by CD4 + T cells and macrophages but also by a substantial presence of CD8 + T cells and NK cells, which may contribute to its less favorable response to these therapies. The varied immune response may require a more individualized approach, potentially incorporating multiple therapeutic strategies to achieve effective disease control. These might be also owing to fewer existing RCTs regarding TAK compared to GCA, which calls for future investigations. Based on the results, we also observed less reduction in glucocorticoids taper dose in cohort studies of TAK. This might be due to the unbalanced baseline usage of glucocorticoids, brought by the nature of observational studies.

Limitations of this review are also obvious. First, the quality of evidence for TAK is extremely poor due to scarcity of double-blind randomized controlled trials examining the effects of biologic agents on TAK. Only two such studies with the outcome of interest were included in this review. Additionally, the six cohort studies on TAK had a moderate to serious risk of bias. In the selection process, only studies reported in English were included in this review, which might also bring limitations. Second, in the cohort studies, unmatched baseline characteristics of the study population and concomitant medications were the main sources of bias, very likely bringing confounding effects on the pooled analysis results and corresponding conclusions. Third, the differences between various biologics were not investigated in this review. Although there was evidence suggesting no significant difference in efficacy between biological agents targeting TNF-α and IL-6 in treatments of TAK as the proportions of complete and partial response at 3, 6, and 12 months were equivalent between groups [33]. Different types of biologic agents with different targets themselves might have different effect sizes for LVV. Overall superiority of them for the treatment of LVV does not guarantee superiority remaining when regarding a specific type of biologic agents. Further analysis comparing the efficacy of different biologic agents is needed.

Taken together, this systematic review and meta-analysis of double-blind RCTs and cohort studies suggested that biologic agents could significantly decrease relapse rates during glucocorticoid tapering, increase remission rate, prolong relapse-free survival, increase glucocorticoid taper rate, decrease cumulative glucocorticoids dose and decrease ESR for GCA and increase glucocorticoid taper rate, decrease cumulative glucocorticoids dose for TAK. Non-significant trends of favoring biologic therapies in reducing CRP level for GCA and relapse rate, ITAS-2010, ITAS-A, ESR, and CRP in TAK and increasing remission rate and relapse-free survival for TAK were also observed. More high-quality RCTs and cohort studies are still needed to bring robust evidence for guiding treatment for LVV patients.

## Supporting information

S1 Fig Effects of biologic agents on relapse rates during glucocorticoid tapering for giant cell arteritis in double-blind randomized controlled trials. The funnel plot (A) and Egger’s test (B) for small-study effects.
(TIF)

S2 Fig Effects of biologic agents on remission rates during glucocorticoid tapering for giant cell arteritis in double-blind randomized controlled trials. The funnel plot (A) and Egger’s test (B) for small-study effects.
(TIF)

S3 Fig Effects of biologic agents on relapse-free survival during glucocorticoid tapering for giant cell arteritis in double-blind randomized controlled trials. The funnel plot (A) and Egger’s test (B) for small-study effects.
(TIF)

S4 Fig Effects of biologic agents on glucocorticoid taper rate for giant cell arteritis in double-blind randomized controlled trials. The funnel plot (A) and Egger’s test (B) for small-study effects.
(TIF)

S5 Fig Effects of biologic agents on cumulative glucocorticoids dose during glucocorticoid tapering for giant cell arteritis in double-blind randomized controlled trials. The funnel plot (A) and Egger’s test (B) for small-study effects.
(TIF)

S6 Fig Effects of biologic agents on changes in ESR for giant cell arteritis in double-blind randomized controlled trials. The forest plot (A), the funnel plot (B) and Egger’s test (C) for small-study effects. SCK =  secukinumab.
(TIF)

S7 Fig Effects of biologic agents on changes in CRP for giant cell arteritis in double-blind randomized controlled trials. The forest plot (A), the funnel plot (B) and Egger’s test (C) for small-study effects. SCK =  secukinumab.
(TIF)

S8 Fig Effects of biologic agents on relapse rate for Takayasu’s arteritis patients in double-blind randomized controlled trials. The forest plot (A), the funnel plot (B) and Egger’s test (C) for small-study effects. ABA =  abatacept; TCZ =  tocilizumab.
(TIF)

S9 Fig Effects of biologic agents on relapse-free survival for Takayasu’s arteritis patients in double-blind randomized controlled trials. The forest plot (A), the funnel plot (B) and Egger’s test (C) for small-study effects. ABA =  abatacept; TCZ =  tocilizumab.
(TIF)

S10 Fig Effects of biologic agents on relapse rate during glucocorticoid tapering for Takayasu’s arteritis in cohort studies. The funnel plot (A) and Egger’s test (B) for small-study effects.
(TIF)

S11 Fig Effects of biologic agents on remission rate during glucocorticoid tapering for Takayasu’s arteritis in cohort studies. The funnel plot (A) and Egger’s test (B) for small-study effects.
(TIF)

S12 Fig Effects of biologic agents on relapse-free survival during glucocorticoid tapering for Takayasu’s arteritis in cohort studies. The funnel plot (A) and Egger’s test (B) for small-study effects.
(TIF)

S13 Fig Effects of biologic agents on glucocorticoid taper rate for Takayasu’s arteritis in cohort studies. The funnel plot (A) and Egger’s test (B) for small-study effects.
(TIF)

S14 Fig Effects of biologic agents on cumulative glucocorticoid dose during glucocorticoid tapering for Takayasu’s arteritis in cohort studies. The funnel plot (A) and Egger’s test (B) for small-study effects.
(TIF)

S15 Fig Effects of biologic agents on changes in glucocorticoid dose for Takayasu’s arteritis patients in cohort studies. The forest plot (A), the funnel plot (B) and Egger’s test (C) for small-study effects. TCZ =  tocilizumab; TOF =  tofacitinib.
(TIF)

S16 Fig Effects of biologic agents on changes in ITAS-2010 for Takayasu’s arteritis patients in cohort studies. The forest plot (A), the funnel plot (B) and Egger’s test (C) for small-study effects. TCZ =  tocilizumab.
(TIF)

S17 Fig Effects of biologic agents on changes in ESR for Takayasu’s arteritis patients in cohort studies. The forest plot (A), the funnel plot (B) and Egger’s test (C) for small-study effects. TCZ =  tocilizumab.
(TIF)

S18 Fig Effects of biologic agents on changes in CRP for Takayasu’s arteritis patients in cohort studies. The forest plot (A), the funnel plot (B) and Egger’s test (C) for small-study effects. TCZ =  tocilizumab.
(TIF)

S19 Fig Effects of biologic agents on changes in ITAS-A for Takayasu’s arteritis patients in cohort studies. The forest plot (A), the funnel plot (B) and Egger’s test (C) for small-study effects. TCZ =  tocilizumab.
(TIF)

S1 Table Search Strategy in MEDLINE. (DOCX)

S2 Table Search Strategy in Embase. (DOCX)

S3 Table Search Strategy in Cochrane Central Registry of Controlled Trials (CENTRAL). (DOCX)

S4 Table Extended information of included studies regarding effects of biologic agents for the treatment of large vessel vasculitis. (DOCX)

S5 Table Definitions of the success of achieving glucocorticoid tapering in different studies. (DOCX)

S6 Table Definitions of relapse in different TAK cohort studies. (DOCX)

S7 Table Definitions of remission in different TAK cohort studies. (DOCX)

S1 File Data extraction form. (DOCX)

S2 File All studies identified in the literature search. (XLSX)

S3 File Data extraction information. (XLSX)

S4 File ROB 2.0 for RCT. (XLSX)

S5 File ROBINS-1 for Cohort. (DOCX)
