# Supplementary figures and images for: Biologic therapies for the treatment of large vessel vasculitis: A systematic review and meta-analysis
Source: PLoS One. 2025 Mar 10;20(3):e0314566. doi: 10.1371/journal.pone.0314566 (PMC11893120; doi:10.1371/journal.pone.0314566)

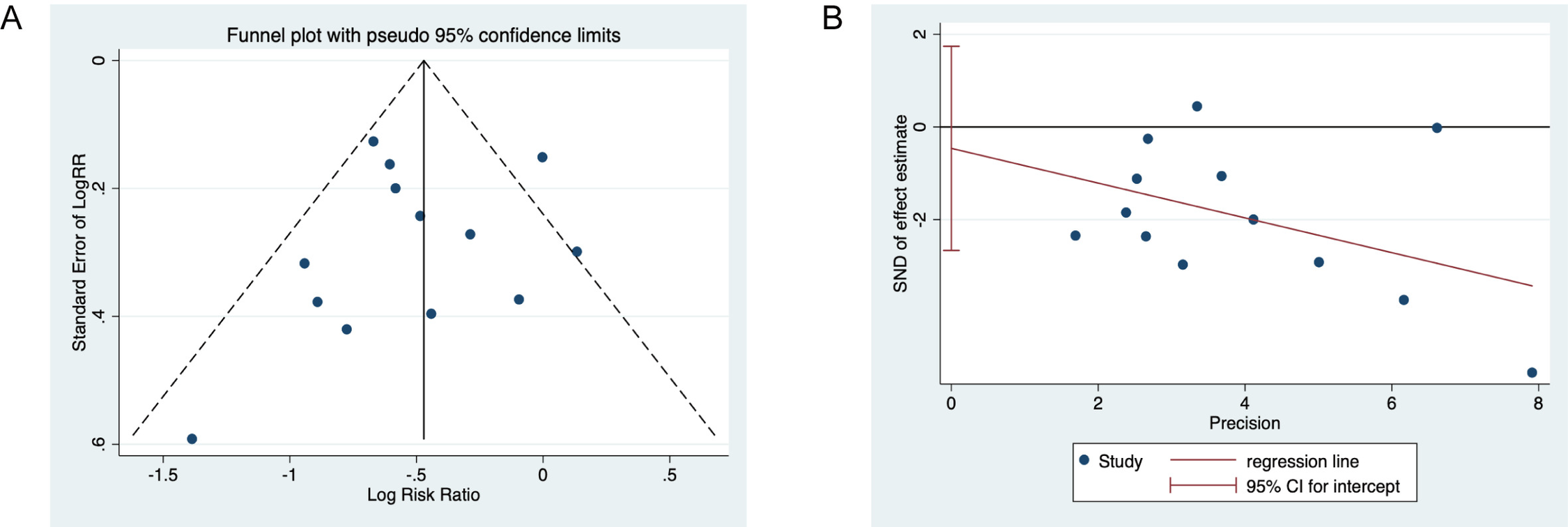

Supplement: S1 Fig — The funnel plot (A) and Egger’s test (B) for small-study effects. (TIF) [file pone.0314566.s001.tif]

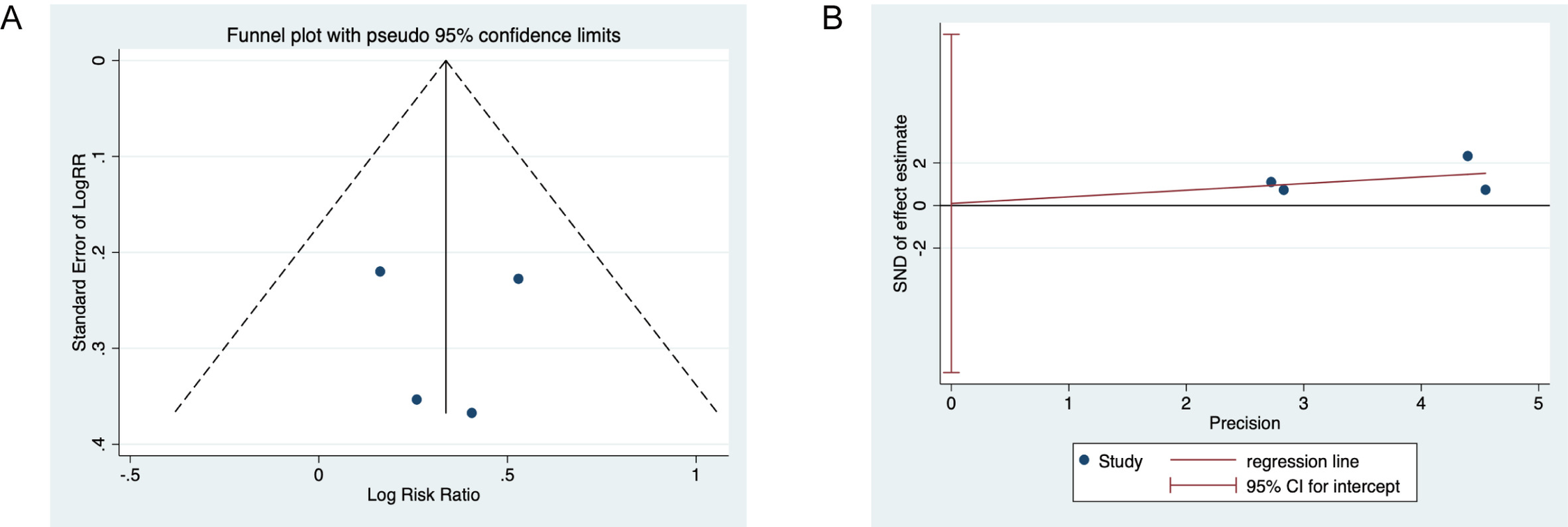

Supplement: S2 Fig — The funnel plot (A) and Egger’s test (B) for small-study effects. (TIF) [file pone.0314566.s002.tif]

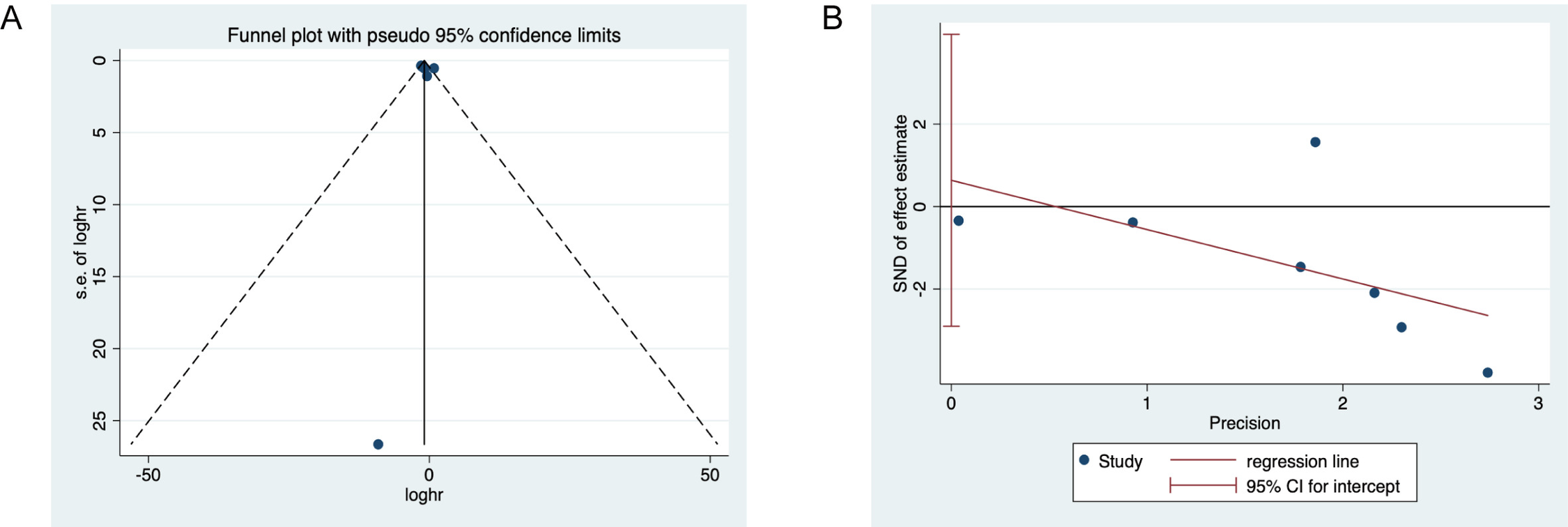

Supplement: S3 Fig — The funnel plot (A) and Egger’s test (B) for small-study effects. (TIF) [file pone.0314566.s003.tif]

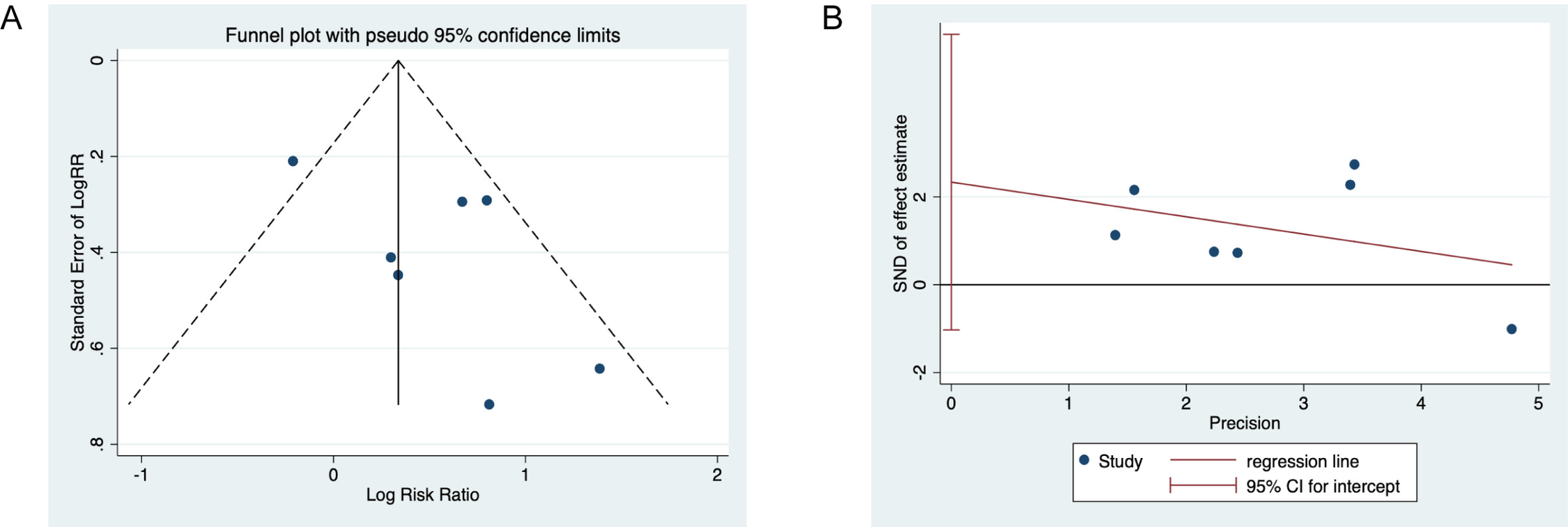

Supplement: S4 Fig — The funnel plot (A) and Egger’s test (B) for small-study effects. (TIF) [file pone.0314566.s004.tif]

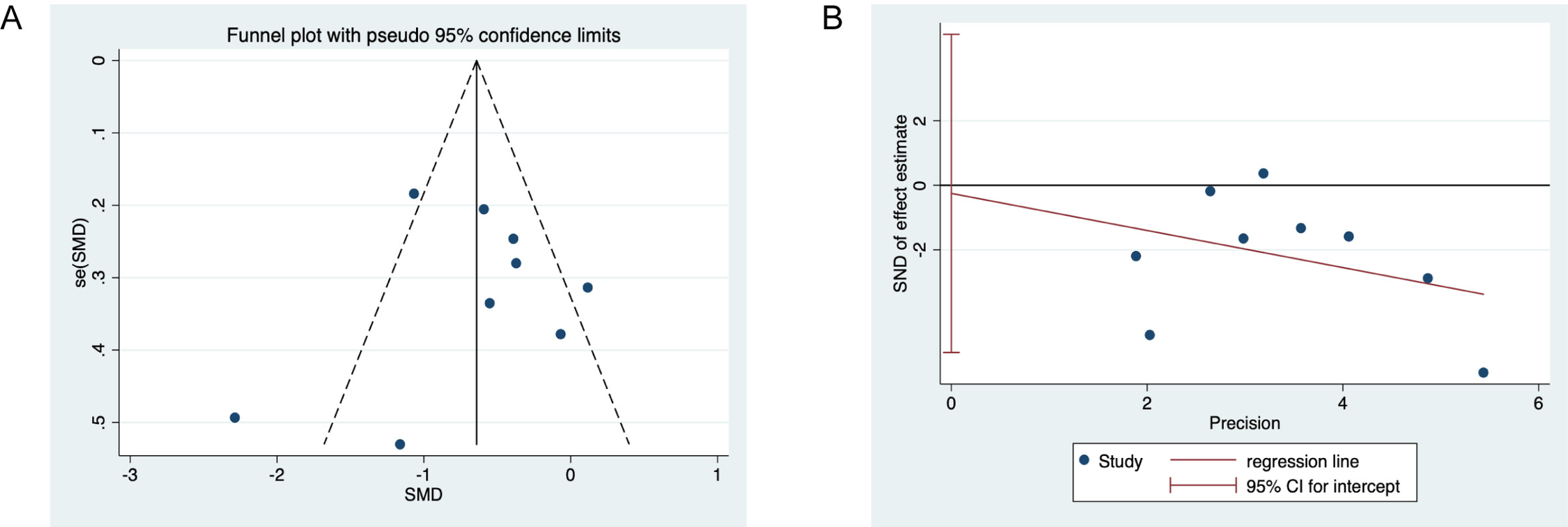

Supplement: S5 Fig — The funnel plot (A) and Egger’s test (B) for small-study effects. (TIF) [file pone.0314566.s005.tif]

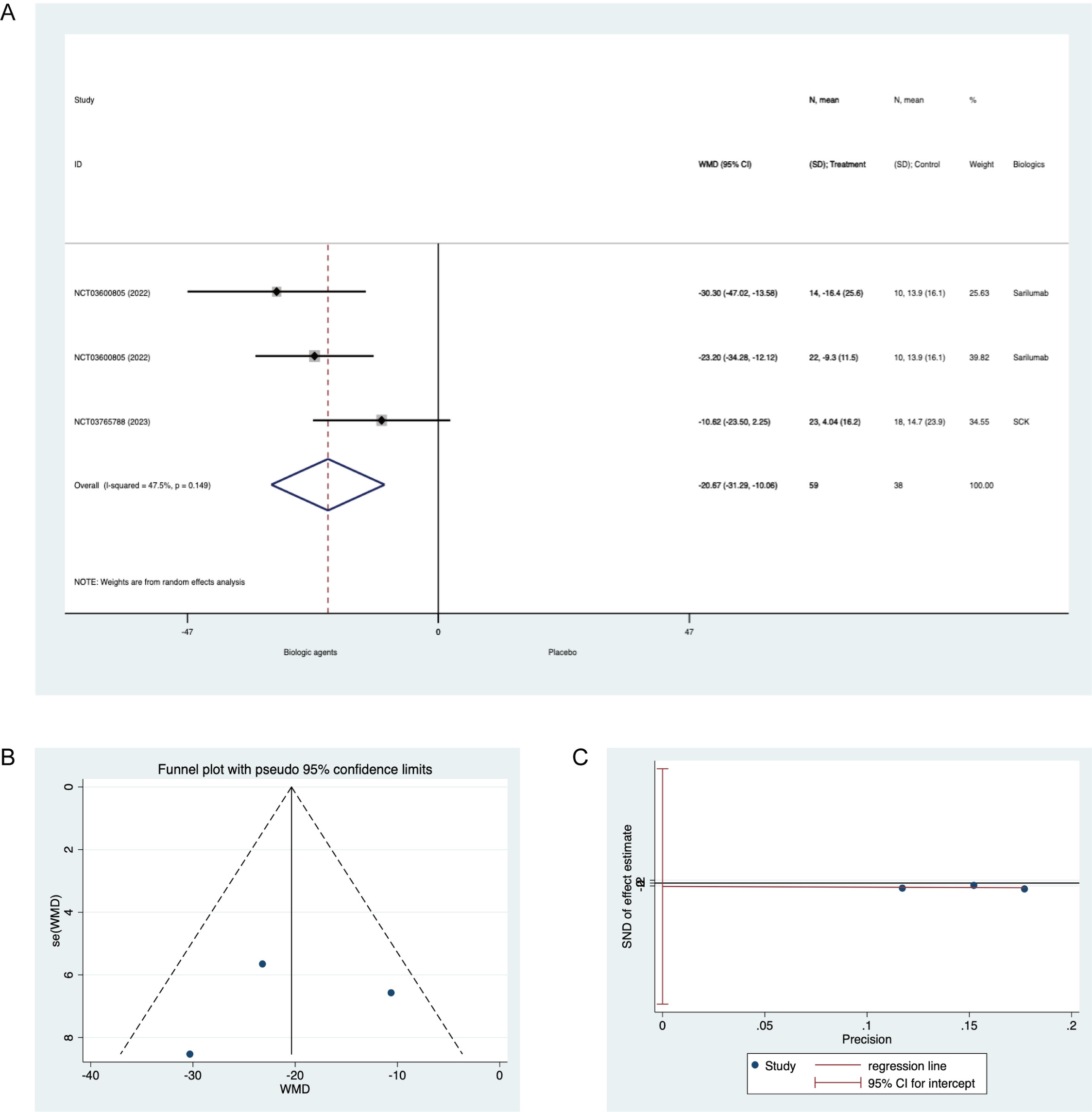

Supplement: S6 Fig — The forest plot (A), the funnel plot (B) and Egger’s test (C) for small-study effects. SCK = secukinumab. (TIF) [file pone.0314566.s006.tif]

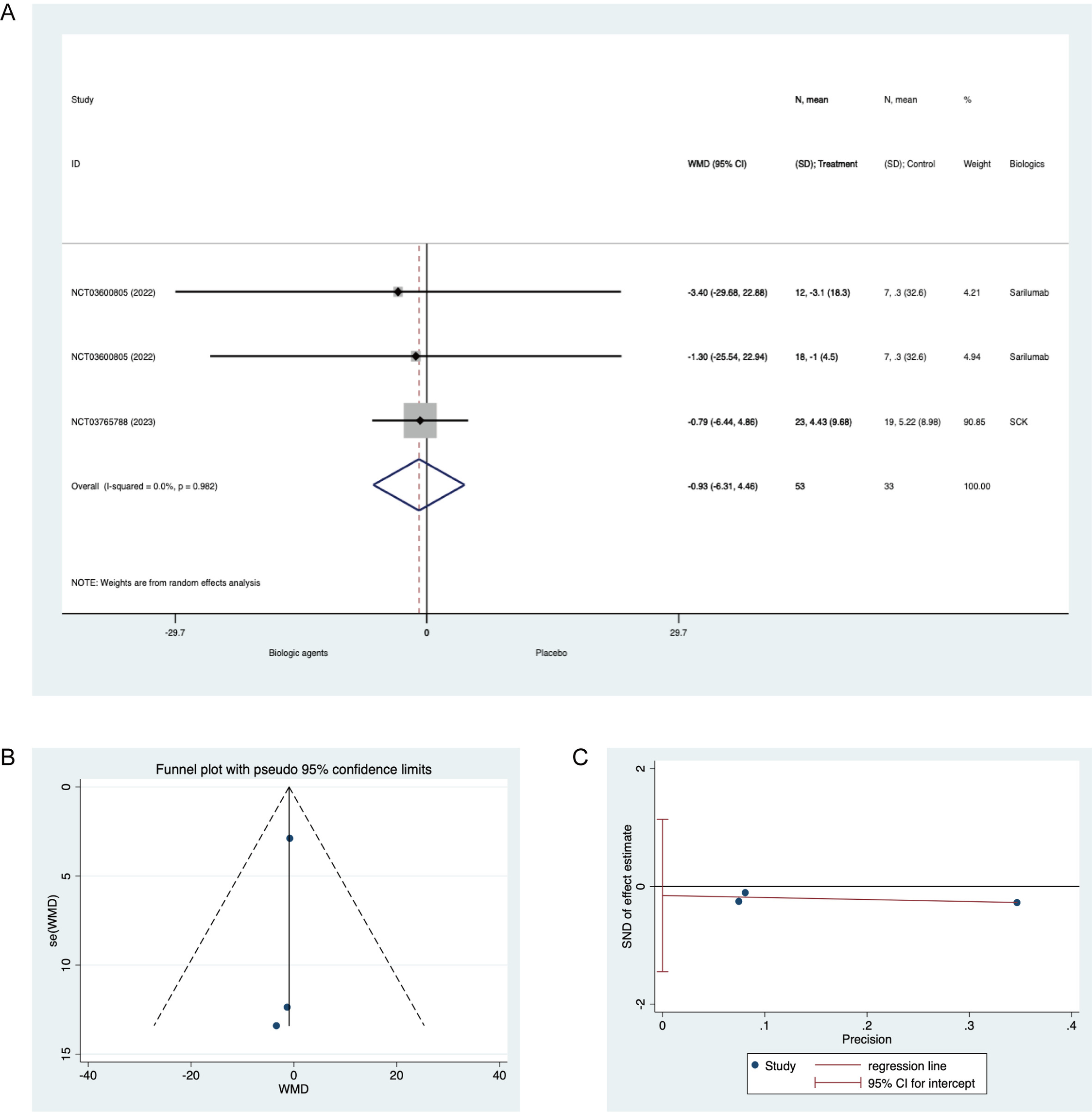

Supplement: S7 Fig — The forest plot (A), the funnel plot (B) and Egger’s test (C) for small-study effects. SCK = secukinumab. (TIF) [file pone.0314566.s007.tif]

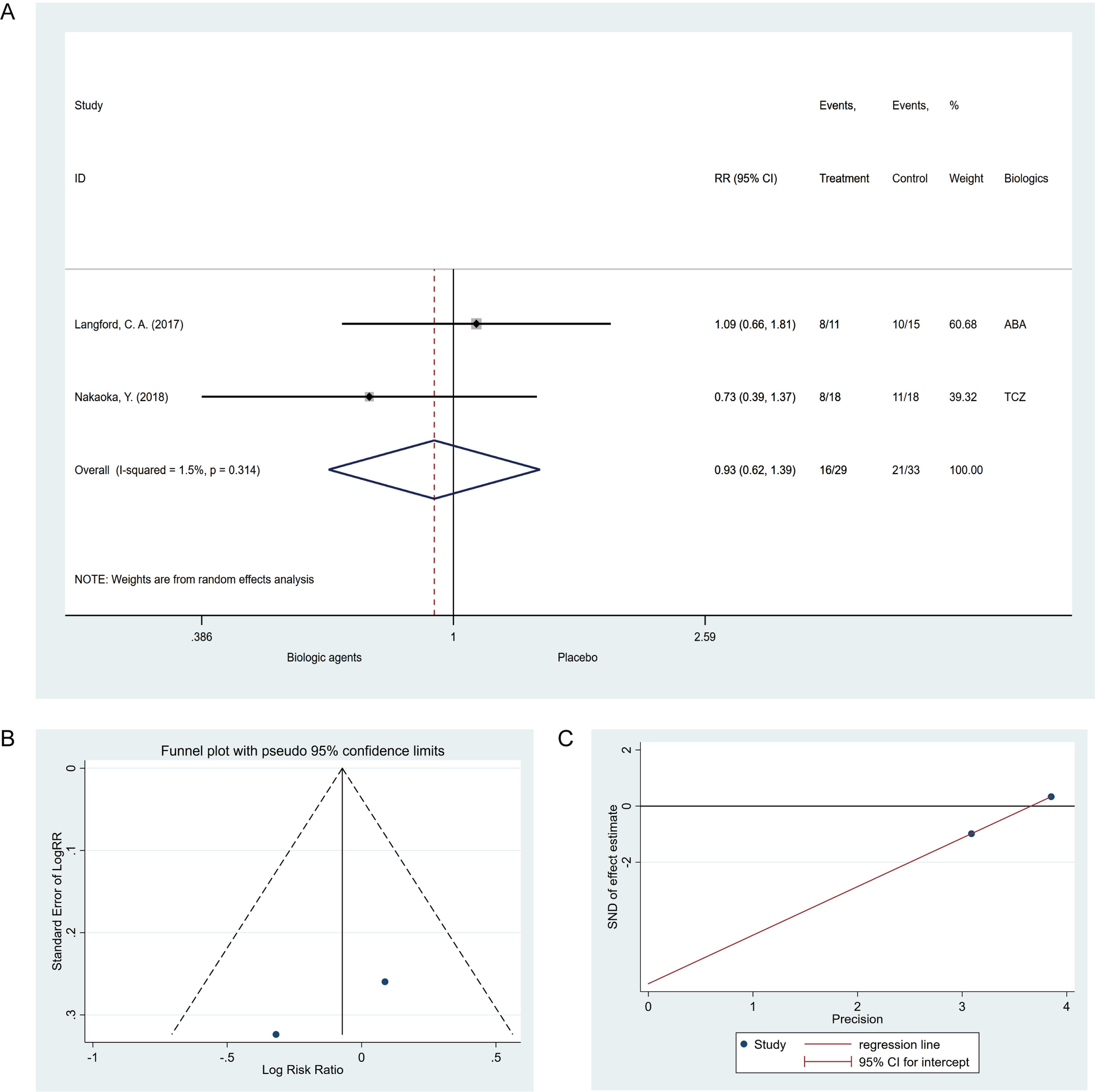

Supplement: S8 Fig — The forest plot (A), the funnel plot (B) and Egger’s test (C) for small-study effects. ABA = abatacept; TCZ = tocilizumab. (TIF) [file pone.0314566.s008.tif]

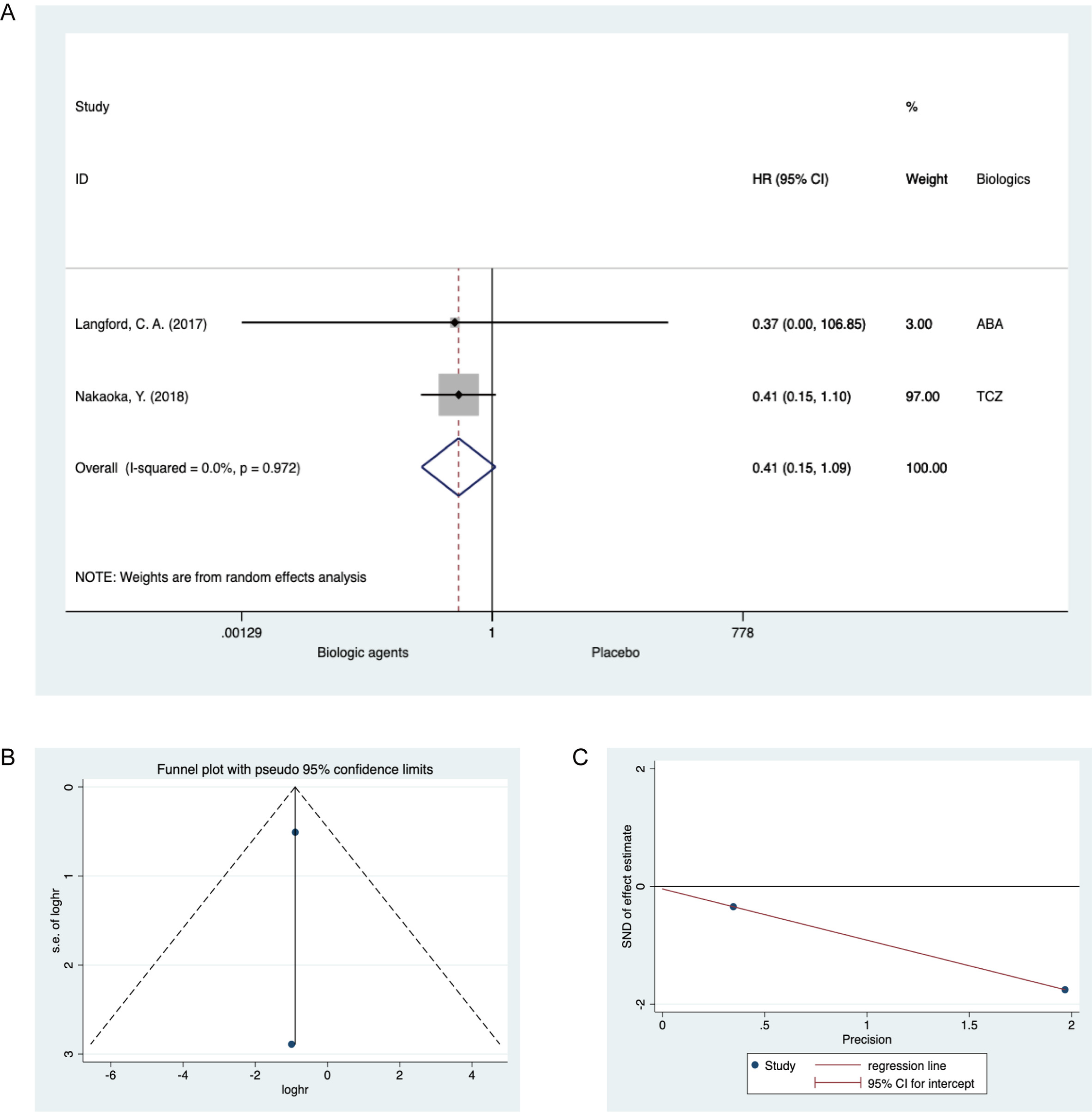

Supplement: S9 Fig — The forest plot (A), the funnel plot (B) and Egger’s test (C) for small-study effects. ABA = abatacept; TCZ = tocilizumab. (TIF) [file pone.0314566.s009.tif]

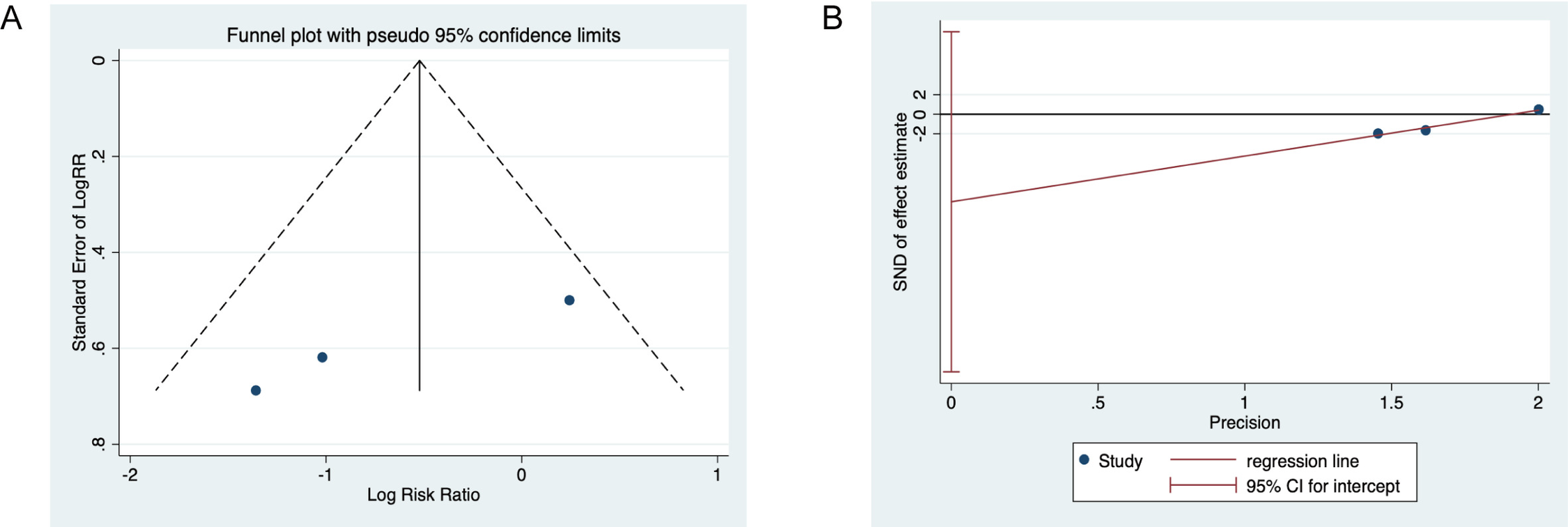

Supplement: S10 Fig — The funnel plot (A) and Egger’s test (B) for small-study effects. (TIF) [file pone.0314566.s010.tif]

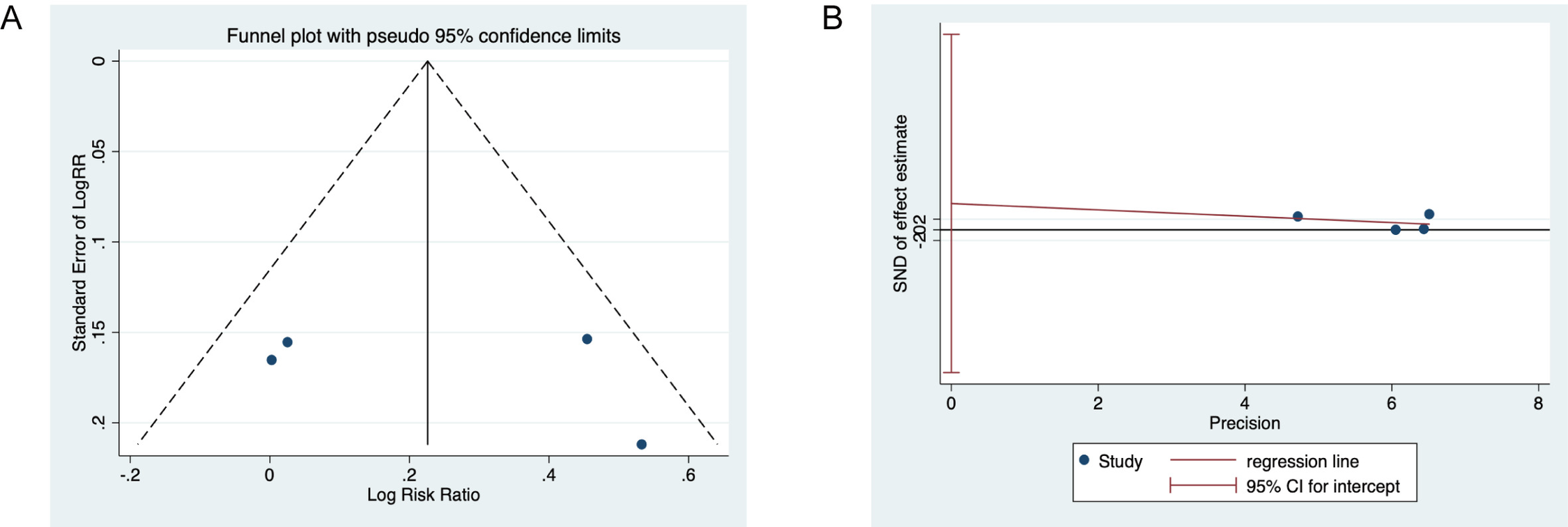

Supplement: S11 Fig — The funnel plot (A) and Egger’s test (B) for small-study effects. (TIF) [file pone.0314566.s011.tif]

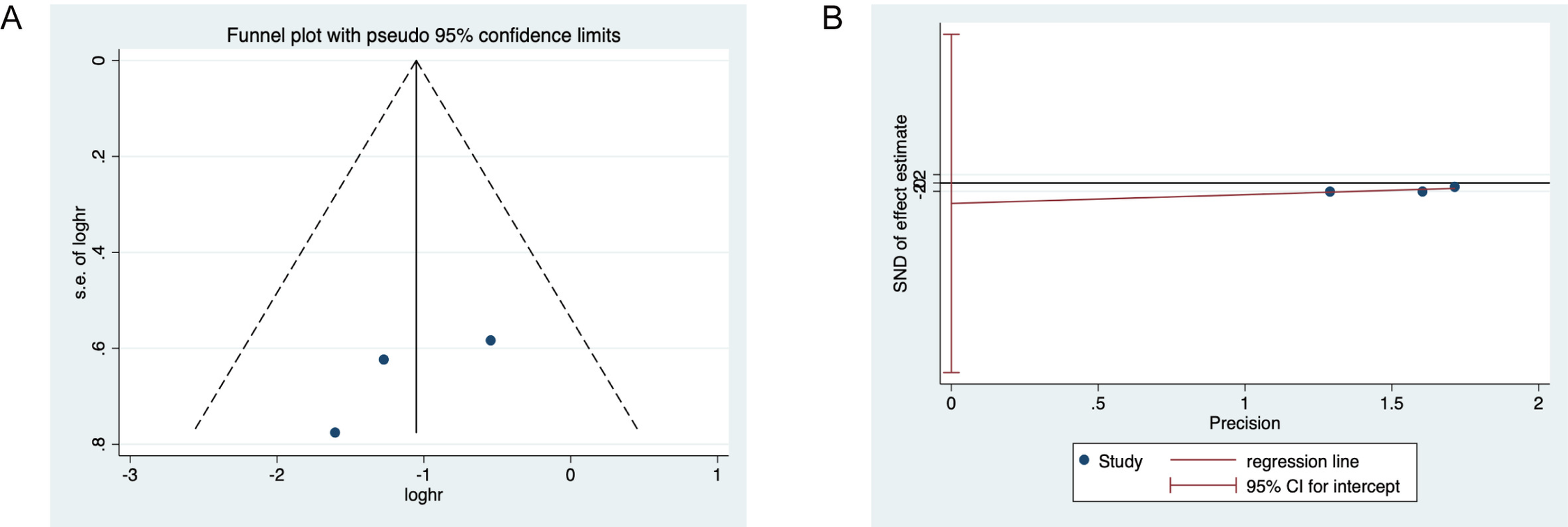

Supplement: S12 Fig — The funnel plot (A) and Egger’s test (B) for small-study effects. (TIF) [file pone.0314566.s012.tif]

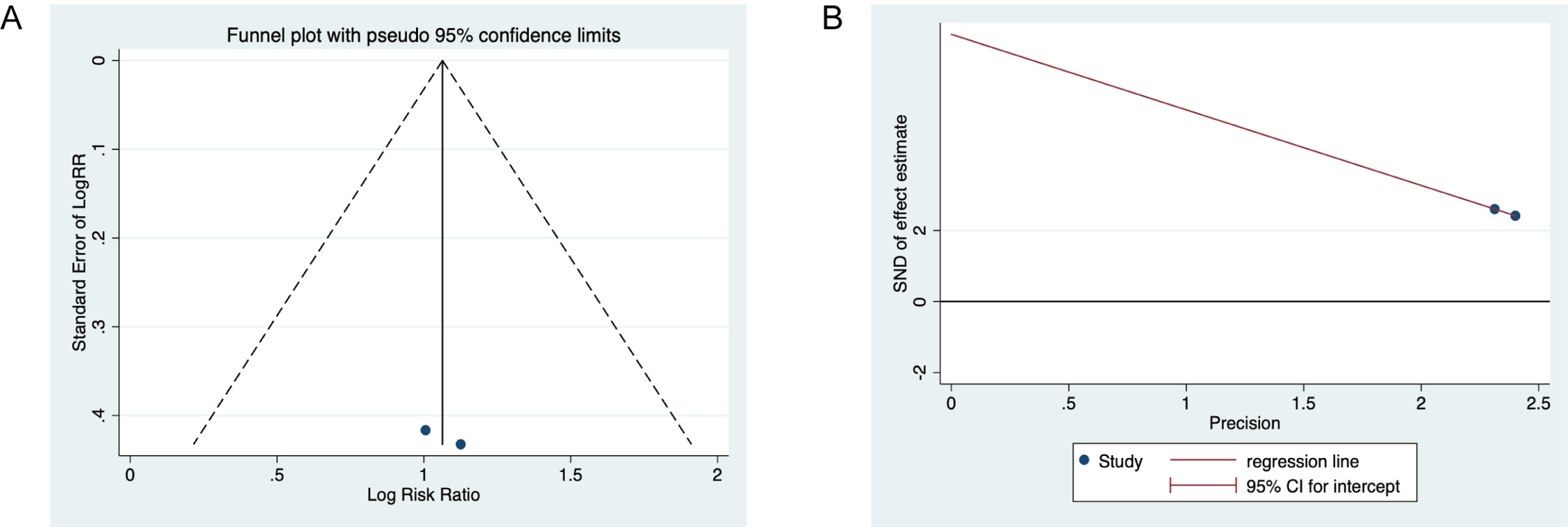

Supplement: S13 Fig — The funnel plot (A) and Egger’s test (B) for small-study effects. (TIF) [file pone.0314566.s013.tif]

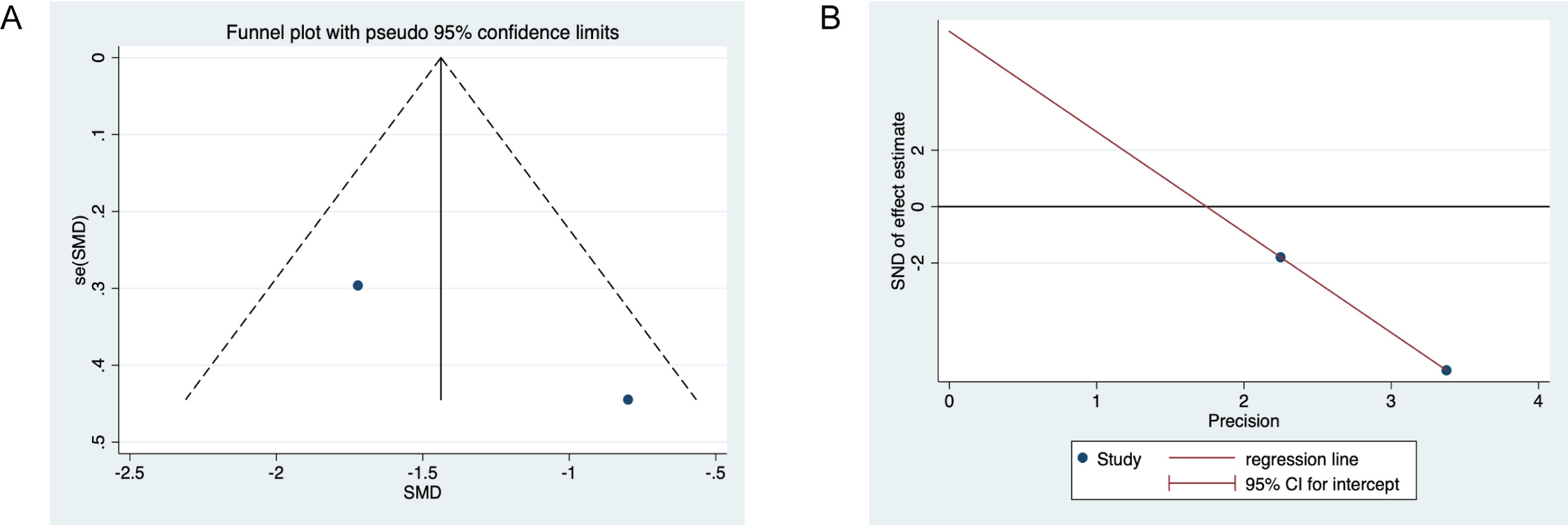

Supplement: S14 Fig — The funnel plot (A) and Egger’s test (B) for small-study effects. (TIF) [file pone.0314566.s014.tif]

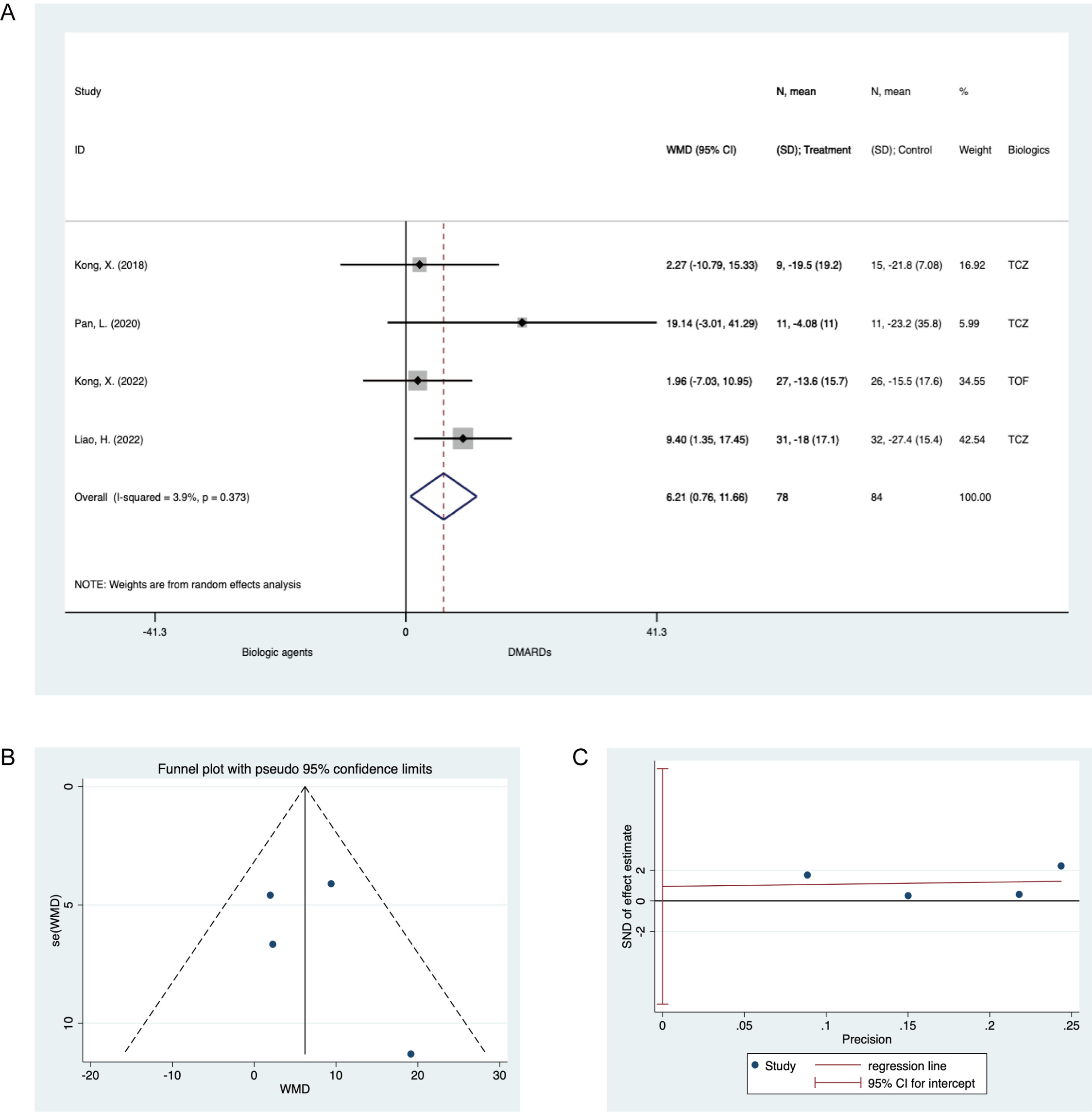

Supplement: S15 Fig — The forest plot (A), the funnel plot (B) and Egger’s test (C) for small-study effects. TCZ = tocilizumab; TOF = tofacitinib. (TIF) [file pone.0314566.s015.tif]

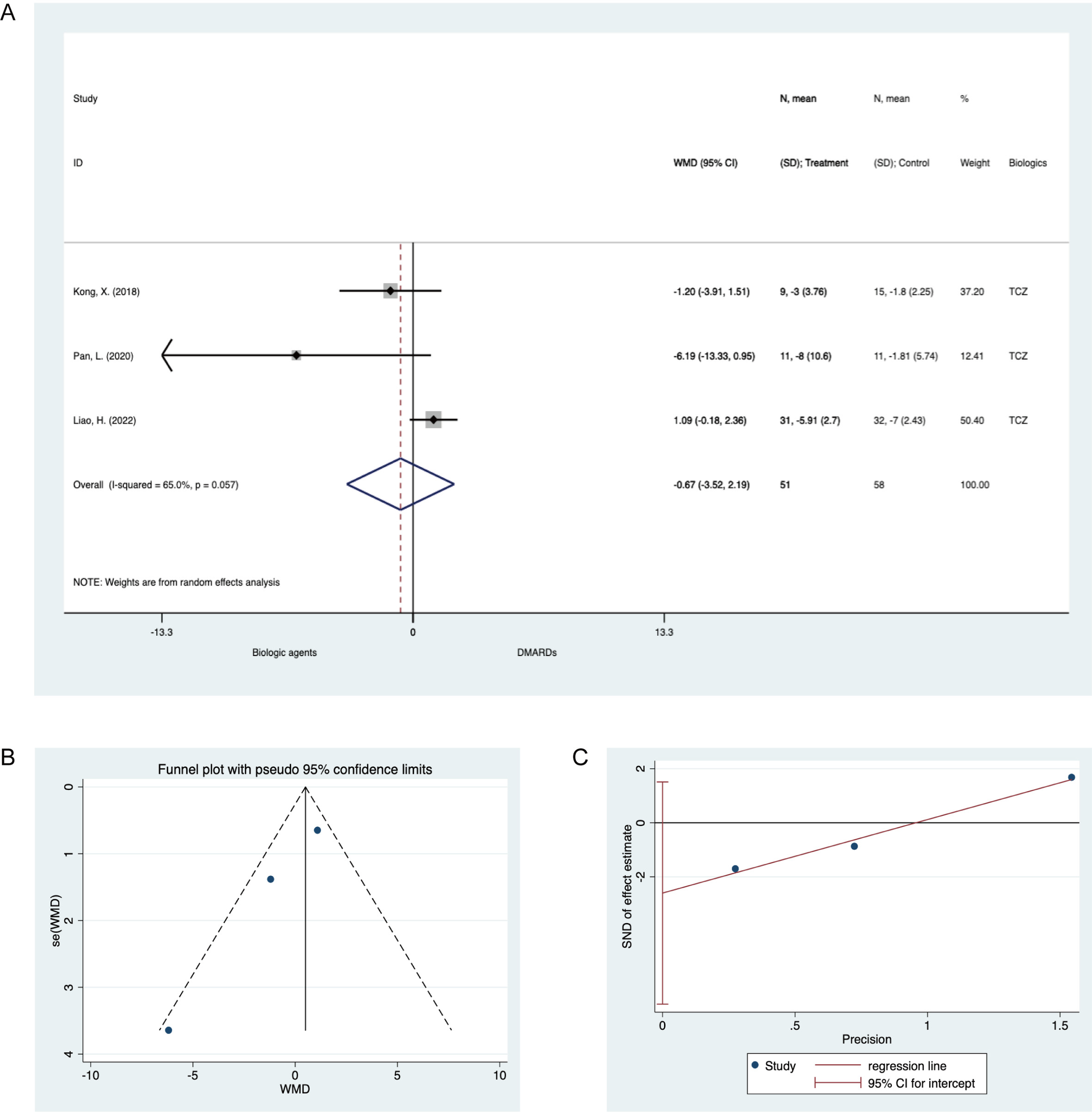

Supplement: S16 Fig — The forest plot (A), the funnel plot (B) and Egger’s test (C) for small-study effects. TCZ = tocilizumab. (TIF) [file pone.0314566.s016.tif]

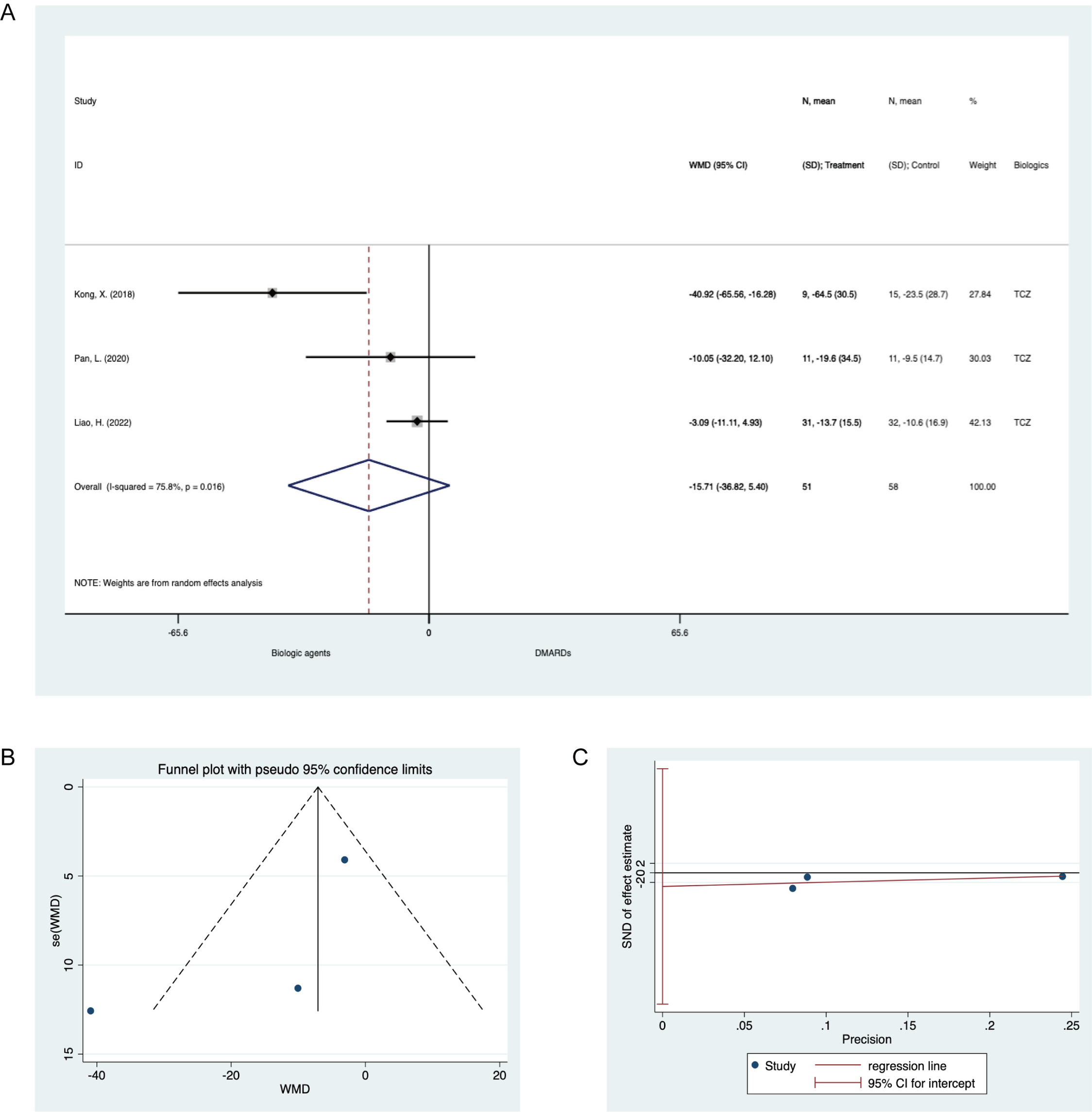

Supplement: S17 Fig — The forest plot (A), the funnel plot (B) and Egger’s test (C) for small-study effects. TCZ = tocilizumab. (TIF) [file pone.0314566.s017.tif]

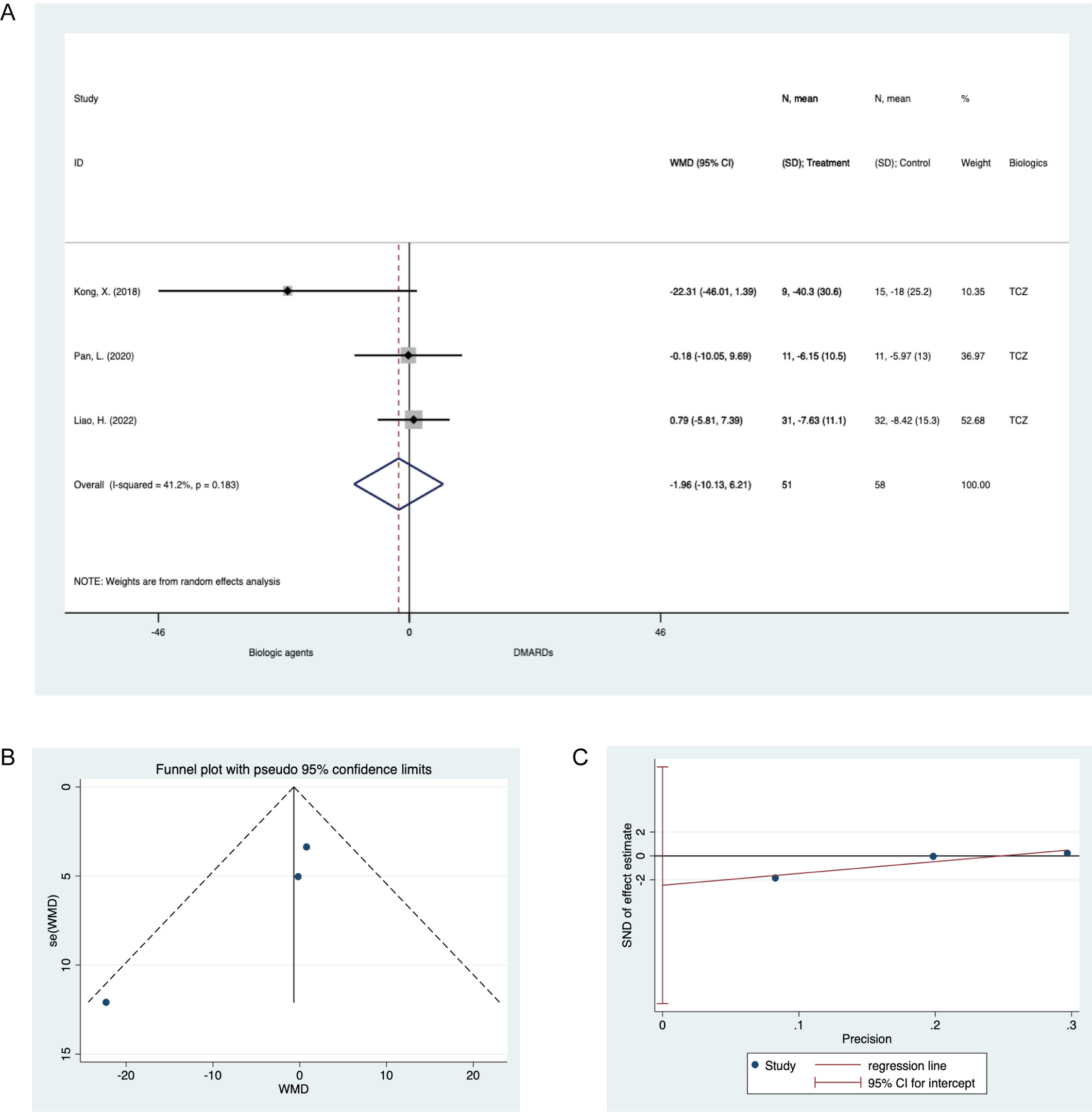

Supplement: S18 Fig — The forest plot (A), the funnel plot (B) and Egger’s test (C) for small-study effects. TCZ = tocilizumab. (TIF) [file pone.0314566.s018.tif]

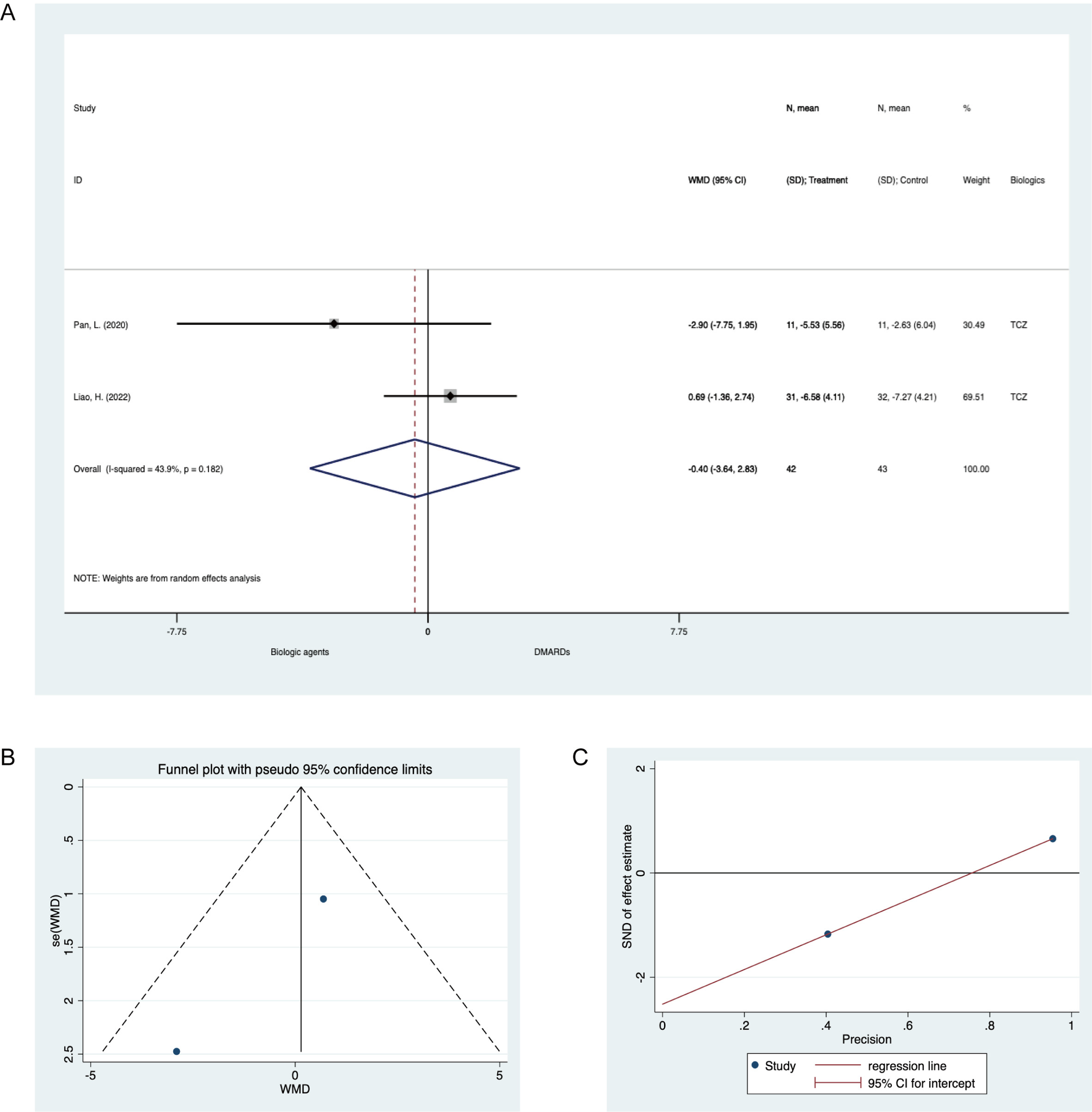

Supplement: S19 Fig — The forest plot (A), the funnel plot (B) and Egger’s test (C) for small-study effects. TCZ = tocilizumab. (TIF) [file pone.0314566.s019.tif]
