# Supplementary material for: Biologic therapies for the treatment of large vessel vasculitis: A systematic review and meta-analysis
Source: PLoS One. 2025 Mar 10;20(3):e0314566. doi: 10.1371/journal.pone.0314566 (PMC11893120; doi:10.1371/journal.pone.0314566)
Supplement: S1 Table — (DOCX) [file pone.0314566.s020.docx]

**S1 Table. Search Strategy in MEDLINE.**

|  |  | **Searching Terms** |
| --- | --- | --- |
| **Population** | #1 | vasculitis[Title/Abstract] OR arteritis[Title/Abstract] |
|  | #2 | "large vessel"[Title/Abstract] |
|  | #3 | #1 AND #2 |
|  | #4 | Takayasu Arteritis [Mesh] |
|  | #5 | Takayasu*[Title/Abstract] |
|  | #6 | Giant Cell Arteritis [Mesh] |
|  | #7 | ("giant cell"[Title/Abstract]) OR (temporal[Title/Abstract]) |
|  | #8 | #1 AND #7 |
|  | #9 | #3 OR #4 OR #5 OR #6 OR #8 |
| **Intervention** | #10 | biologic*[Title/Abstract] |
|  | #11 | anti*[Title/Abstract] OR monoclonal[Title/Abstract] OR immuno*[Title/Abstract] OR Ig[Title/Abstract] OR inhibitor*[Title/Abstract] OR antagonist*[Title/Abstract] OR block*[Title/Abstract] OR against[Title/Abstract] OR agonist*[Title/Abstract] OR stimul*[Title/Abstract] |
|  | #12 | interleukin*[Title/Abstract] OR "IL-1"[Title/Abstract] OR "IL-6"[Title/Abstract] OR "IL-12*"[Title/Abstract] OR "IL-23*"[Title/Abstract] OR "IL-17*"[Title/Abstract] OR IL1[Title/Abstract] OR IL6[Title/Abstract] OR IL12[Title/Abstract] OR IL23[Title/Abstract] OR IL17[Title/Abstract] OR "tumor necrosis factor*"[Title/Abstract] OR TNF[Title/Abstract] OR "cytotoxic T lymphocyte associated antigen*"[Title/Abstract] OR CTLA[Title/Abstract] OR p40[Title/Abstract] OR CD20[Title/Abstract] OR "CD-20*"[Title/Abstract] OR JAK[Title/Abstract] OR "janus kinase*"[Title/Abstract] |
|  | #13 | ("Abatacept"[Mesh]) OR (Abatacept*[Title/Abstract] OR LEA29Y[Title/Abstract] OR "BMS-224818"[Title/Abstract] OR BMS224818[Title/Abstract] OR Belatacept[Title/Abstract] OR Orencia[Title/Abstract] OR "BMS-188667"[Title/Abstract] OR BMS188667[Title/Abstract] OR Nulojix[Title/Abstract]) |
|  | #14 | ("Adalimumab"[Mesh]) OR (Adalimumab*[Title/Abstract] OR Humira[Title/Abstract] OR Amjevita[Title/Abstract] OR Cyltezo[Title/Abstract] OR D2E7[Title/Abstract]) |
|  | #15 | ("Interleukin 1 Receptor Antagonist Protein"[Mesh]) OR (Anakinra*[Title/Abstract] OR Febrile[Title/Abstract] OR Antril[Title/Abstract] OR Kineret[Title/Abstract]) |
|  | #16 | (baricitinib [Supplementary Concept]) OR (baricitinib*[Title/Abstract] OR LY3009104[Title/Abstract] OR Olumiant[Title/Abstract] OR INCB028050[Title/Abstract]) |
|  | #17 | ("Certolizumab Pegol"[Mesh]) OR (Certolizumab*[Title/Abstract] OR Cimzia[Title/Abstract] OR "CDP-870"[Title/Abstract] OR CDP870[Title/Abstract]) |
|  | #18 | ("Etanercept"[Mesh]) OR (etanercept*[Title/Abstract] OR TNFR*[Title/Abstract] OR TNR[Title/Abstract] OR TNT[Title/Abstract] OR TNTR*[Title/Abstract] OR Erelzi[Title/Abstract] OR Enbrel[Title/Abstract]) |
|  | #19 | ("gevokizumab" [Supplementary Concept]) OR (gevokizumab*[Title/Abstract] OR "XMA-005.2"[Title/Abstract] OR XMA005.2[Title/Abstract] OR "XOMA-052"[Title/Abstract] OR XOMA052[Title/Abstract]) |
|  | #20 | ("golimumab" [Supplementary Concept]) OR (golimumab*[Title/Abstract] OR "CNTO-148"[Title/Abstract] OR CNTO148[Title/Abstract] OR Simponi[Title/Abstract]) |
|  | #21 | ("guselkumab" [Supplementary Concept]) OR (guselkumab*[Title/Abstract] OR Tremfya[Title/Abstract] OR "CNTO-1959"[Title/Abstract] OR CNTO1959[Title/Abstract]) |
|  | #22 | ("Infliximab"[Mesh]) OR (Infliximab*[Title/Abstract] OR cA2[Title/Abstract] OR Renflexis[Title/Abstract] OR Inflectra[Title/Abstract] OR Remicade[Title/Abstract]) |
|  | #23 | ("mavrilimumab" [Supplementary Concept]) OR (mavrilimumab*[Title/Abstract] OR "CAM-3001"[Title/Abstract] OR CAM3001[Title/Abstract]) |
|  | #24 | ("Rituximab"[Mesh]) OR (Rituximab*[Title/Abstract] OR Mabthera[Title/Abstract] OR "IDEC-C2B8"[Title/Abstract] OR GP2013[Title/Abstract] OR Rituxan[Title/Abstract]) |
|  | #25 | ("sarilumab" [Supplementary Concept]) OR (sarilumab*[Title/Abstract] OR "SAR-153191"[Title/Abstract] OR SAR153191[Title/Abstract] OR Kevzara[Title/Abstract] OR "REGN-88"[Title/Abstract] OR REGN88[Title/Abstract]) |
|  | #26 | ("secukinumab" [Supplementary Concept]) OR (secukinumab*[Title/Abstract] OR Cosentyx[Title/Abstract] OR "AIN-457"[Title/Abstract] OR AIN457[Title/Abstract]) |
|  | #27 | ("sirukumab" [Supplementary Concept]) OR (sirukumab*[Title/Abstract] OR "CNTO-136"[Title/Abstract] OR CNTO136[Title/Abstract]) |
|  | #28 | ("tocilizumab" [Supplementary Concept]) OR (tocilizumab*[Title/Abstract] OR "RHPM-1"[Title/Abstract] OR RHPM1[Title/Abstract] OR "RG-1569"[Title/Abstract] OR RG1569[Title/Abstract] OR R-1569[Title/Abstract] OR R1569[Title/Abstract] OR "MSB-11456"[Title/Abstract] OR MSB11456[Title/Abstract] OR atlizumab[Title/Abstract] OR MRA[Title/Abstract] OR "RO-4877533"[Title/Abstract] OR RO4877533[Title/Abstract] OR Actemra[Title/Abstract] OR Roactemra[Title/Abstract]) |
|  | #29 | ("upadacitinib" [Supplementary Concept]) OR (upadacitinib*[Title/Abstract] OR "ABT-494"[Title/Abstract] OR ABT494[Title/Abstract] OR Rinvoq[Title/Abstract]) |
|  | #30 | ("Ustekinumab"[Mesh]) OR (ustekinumab*[Title/Abstract] OR Stelara[Title/Abstract] OR "CNTO-1275"[Title/Abstract] OR CNTO1275[Title/Abstract]) |
|  | #31 | ("tofacitinib" [Supplementary Concept]) OR (tofacitinib*[Title/Abstract] OR tasocitinib[Title/Abstract] OR Xeljanz[Title/Abstract] OR "CP-690,550"[Title/Abstract] OR CP690550[Title/Abstract] OR "CP-690550"[Title/Abstract]) |
|  | #32 | #10 OR #11 OR #12 OR #13 OR #14 OR #15 OR #16 OR #17 OR #18 OR #19 OR #20 OR #21 OR #22 OR #23 OR #24 OR #25 OR #26 OR #27 OR #28 OR #29 OR #30 OR #31 |
| **Study Design** | #33 | cohort*[Title/Abstract] OR retrospective*[Title/Abstract] OR prospective*[Title/Abstract] OR observational[Title/Abstract] |
|  | #34 | randomi*[Title/Abstract] OR control*[Title/Abstract] OR trial*[Title/Abstract] |
|  | #35 | #33 OR #34 |
