# Supplementary material for: Biologic therapies for the treatment of large vessel vasculitis: A systematic review and meta-analysis
Source: PLoS One. 2025 Mar 10;20(3):e0314566. doi: 10.1371/journal.pone.0314566 (PMC11893120; doi:10.1371/journal.pone.0314566)
Supplement: S2 Table — (DOCX) [file pone.0314566.s021.docx]

**S2 Table. Search Strategy in Embase.**

|  |  | **Search Terms** |
| --- | --- | --- |
| **Population** | #1 | vasculitis:ab,ti OR arteritis:ab,ti |
|  | #2 | 'large vessel vasculitis'/exp |
|  | #3 | 'large vessel':ab,ti |
|  | #4 | #1 AND #3 |
|  | #5 | 'aortic arch syndrome'/exp |
|  | #6 | takayasu*:ab,ti |
|  | #7 | 'giant cell arteritis'/exp |
|  | #8 | 'giant cell':ab,ti OR temporal:ab,ti |
|  | #9 | #1 AND #8 |
|  | #10 | #2 OR #4 OR #5 OR #6 OR #7 OR #9 |
| **Intervention** | #11 | biologic*:ab,ti |
|  | #12 | anti*:ab,ti OR monoclonal:ab,ti OR immuno*:ab,ti OR ig:ab,ti OR inhibitor*:ab,ti OR antagonist*:ab,ti OR block*:ab,ti OR against:ab,ti OR agonist*:ab,ti OR stimul*:ab,ti |
|  | #13 | interleukin*:ab,ti OR 'il-1':ab,ti OR 'il-6':ab,ti OR 'il-12*':ab,ti OR 'il-23*':ab,ti OR 'il-17*':ab,ti OR il1:ab,ti OR il6:ab,ti OR il12:ab,ti OR il23:ab,ti OR il17:ab,ti OR 'tumor necrosis factor*':ab,ti OR tnf:ab,ti OR 'cytotoxic t lymphocyte associated antigen*':ab,ti OR ctla:ab,ti OR p40:ab,ti OR cd20:ab,ti OR 'cd-20*':ab,ti OR jak:ab,ti OR 'janus kinase*':ab,ti |
|  | #14 | 'abatacept'/exp |
|  | #15 | abatacept*:ab,ti OR lea29y:ab,ti OR 'bms-224818':ab,ti OR bms224818:ab,ti OR belatacept:ab,ti OR orencia:ab,ti OR 'bms-188667':ab,ti OR bms188667:ab,ti OR nulojix:ab,ti |
|  | #16 | 'adalimumab'/exp |
|  | #17 | adalimumab*:ab,ti OR humira:ab,ti OR amjevita:ab,ti OR cyltezo:ab,ti OR d2e7:ab,ti |
|  | #18 | 'anakinra'/exp |
|  | #19 | anakinra*:ab,ti OR febrile:ab,ti OR antril:ab,ti OR kineret:ab,ti |
|  | #20 | 'baricitinib'/exp |
|  | #21 | baricitinib*:ab,ti OR ly3009104:ab,ti OR olumiant:ab,ti OR incb028050:ab,ti |
|  | #22 | 'certolizumab pegol'/exp |
|  | #23 | certolizumab*:ab,ti OR cimzia:ab,ti OR 'cdp-870':ab,ti OR cdp870:ab,ti |
|  | #24 | 'etanercept'/exp |
|  | #25 | etanercept*:ab,ti OR tnfr*:ab,ti OR tnr:ab,ti OR tnt:ab,ti OR tntr*:ab,ti OR erelzi:ab,ti OR enbrel:ab,ti |
|  | #26 | 'gevokizumab'/exp |
|  | #27 | gevokizumab*:ab,ti OR 'xma-005.2':ab,ti OR xma005.2:ab,ti OR 'xoma-052':ab,ti OR xoma052:ab,ti |
|  | #28 | 'golimumab'/exp |
|  | #29 | golimumab*:ab,ti OR 'cnto-148':ab,ti OR cnto148:ab,ti OR simponi:ab,ti |
|  | #30 | 'guselkumab'/exp |
|  | #31 | guselkumab*:ab,ti OR tremfya:ab,ti OR 'cnto-1959':ab,ti OR cnto1959:ab,ti |
|  | #32 | 'infliximab'/exp |
|  | #33 | infliximab*:ab,ti OR ca2:ab,ti OR renflexis:ab,ti OR inflectra:ab,ti OR remicade:ab,ti |
|  | #34 | 'mavrilimumab'/exp |
|  | #35 | mavrilimumab*:ab,ti OR 'cam-3001':ab,ti OR cam3001:ab,ti |
|  | #36 | 'rituximab'/exp |
|  | #37 | rituximab*:ab,ti OR mabthera:ab,ti OR 'idec-c2b8':ab,ti OR gp2013:ab,ti OR rituxan:ab,ti |
|  | #38 | 'sarilumab'/exp |
|  | #39 | sarilumab*:ab,ti OR 'sar-153191':ab,ti OR sar153191:ab,ti OR kevzara:ab,ti OR 'regn-88':ab,ti OR regn88:ab,ti |
|  | #40 | 'secukinumab'/exp |
|  | #41 | secukinumab*:ab,ti OR cosentyx:ab,ti OR 'ain-457':ab,ti OR ain457:ab,ti |
|  | #42 | 'sirukumab'/exp |
|  | #43 | sirukumab*:ab,ti OR 'cnto-136':ab,ti OR cnto136:ab,ti |
|  | #44 | 'tocilizumab'/exp |
|  | #45 | tocilizumab*:ab,ti OR 'rhpm-1':ab,ti OR rhpm1:ab,ti OR 'rg-1569':ab,ti OR rg1569:ab,ti OR 'r 1569':ab,ti OR r1569:ab,ti OR 'msb-11456':ab,ti OR msb11456:ab,ti OR atlizumab:ab,ti OR mra:ab,ti OR 'ro-4877533':ab,ti OR ro4877533:ab,ti OR actemra:ab,ti OR roactemra:ab,ti |
|  | #46 | 'upadacitinib'/exp |
|  | #47 | upadacitinib*:ab,ti OR 'abt-494':ab,ti OR abt494:ab,ti OR rinvoq:ab,ti |
|  | #48 | 'ustekinumab'/exp |
|  | #49 | ustekinumab*:ab,ti OR stelara:ab,ti OR 'cnto-1275':ab,ti OR cnto1275:ab,ti |
|  | #50 | 'tofacitinib'/exp |
|  | #51 | tofacitinib*:ab,ti OR tasocitinib:ab,ti OR xeljanz:ab,ti OR 'cp-690,550':ab,ti OR cp690550:ab,ti OR 'cp-690550':ab,ti |
|  | #52 | #11 OR #12 OR #13 OR #14 OR #15 OR #16 OR #17 OR #18 OR #19 OR #20 OR #21 OR #22 OR #23 OR #24 OR #25 OR #26 OR #27 OR #28 OR #29 OR #30 OR #31 OR #32 OR #33 OR #34 OR #35 OR #36 OR #37 OR #38 OR #39 OR #40 OR #41 OR #42 OR #43 OR #44 OR #45 OR #46 OR #47 OR #48 OR #49 OR #50 OR #51 |
| **Study Design** | #53 | cohort*:ab,ti OR retrospective*:ab,ti OR prospective*:ab,ti OR observational:ab,ti |
|  | #54 | randomi*:ab,ti OR control*:ab,ti OR trial*:ab,ti |
|  | #55 | #53 OR #54 |
