# Supplementary material for: Biologic therapies for the treatment of large vessel vasculitis: A systematic review and meta-analysis
Source: PLoS One. 2025 Mar 10;20(3):e0314566. doi: 10.1371/journal.pone.0314566 (PMC11893120; doi:10.1371/journal.pone.0314566)
Supplement: S3 Table — (DOCX) [file pone.0314566.s022.docx]

**S3 Table. Search Strategy in Cochrane Central Registry of Controlled Trials (CENTRAL).**

|  |  | **Search Terms** |
| --- | --- | --- |
| **Population** | #1 | (vasculitis OR arteritis):ti,ab,kw |
|  | #2 | (“large vessel”):ti,ab,kw |
|  | #3 | #1 AND #2 |
|  | #4 | MeSH descriptor: [Takayasu Arteritis] explode all trees |
|  | #5 | (Takayasu*):ti,ab,kw |
|  | #6 | MeSH descriptor: [Giant Cell Arteritis] explode all trees |
|  | #7 | (“giant cell”):ti,ab,kw |
|  | #8 | (temporal):ti,ab,kw |
|  | #9 | #7 OR #8 |
|  | #10 | #1 AND #9 |
|  | #11 | #3 OR #4 OR #5 OR #6 OR #10 |
| **Intervention** | #12 | (biologic*):ti,ab,kw |
|  | #13 | (anti* OR monoclonal OR immuno* OR Ig OR inhibitor* OR antagonist* OR block* OR against OR agonist* OR stimul*):ti,ab,kw |
|  | #14 | (interleukin* OR “IL-1” OR “IL-6” OR “IL-12*” OR “IL-23*” OR “IL-17*” OR IL1 OR IL6 OR IL12 OR IL23 OR IL17 OR “tumor necrosis factor*” OR TNF OR “cytotoxic T lymphocyte associated antigen*” OR CTLA OR p40 OR CD20 OR “CD-20*” OR JAK OR “janus kinase*”):ti,ab,kw |
|  | #15 | MeSH descriptor: [Abatacept] explode all trees |
|  | #16 | (Abatacept* OR LEA29Y OR “BMS-224818” OR BMS224818 OR Belatacept OR Orencia OR “BMS-188667” OR BMS188667 OR Nulojix):ti,ab,kw |
|  | #17 | MeSH descriptor: [Adalimumab] explode all trees |
|  | #18 | (Adalimumab* OR Humira OR Amjevita OR Cyltezo OR D2E7):ti,ab,kw |
|  | #19 | MeSH descriptor: [Interleukin 1 Receptor Antagonist Protein] explode all trees |
|  | #20 | (Anakinra* OR Febrile OR Antril OR Kineret):ti,ab,kw |
|  | #21 | (baricitinib* OR LY3009104 OR Olumiant OR INCB028050):ti,ab,kw |
|  | #22 | (Certolizumab* OR Cimzia OR “CDP-870” OR CDP870):ti,ab,kw |
|  | #23 | MeSH descriptor: [Etanercept] explode all trees |
|  | #24 | (etanercept* OR TNFR* OR TNR OR TNT OR TNTR* OR Erelzi OR Enbrel):ti,ab,kw |
|  | #25 | (gevokizumab* OR “XMA-005.2” OR XMA005.2 OR “XOMA-052” OR XOMA052):ti,ab,kw |
|  | #26 | (golimumab* OR “CNTO-148” OR CNTO148 OR Simponi):ti,ab,kw |
|  | #27 | (guselkumab* OR Tremfya OR “CNTO-1959” OR CNTO1959):ti,ab,kw |
|  | #28 | MeSH descriptor: [Infliximab] explode all trees |
|  | #29 | (Infliximab* OR cA2 OR Renflexis OR Inflectra OR Remicade):ti,ab,kw |
|  | #30 | (mavrilimumab* OR “CAM-3001” OR CAM3001):ti,ab,kw |
|  | #31 | MeSH descriptor: [Rituximab] explode all trees |
|  | #32 | (Rituximab* OR Mabthera OR “IDEC-C2B8” OR GP2013 OR Rituxan):ti,ab,kw |
|  | #33 | (sarilumab* OR “SAR-153191” OR SAR153191 OR Kevzara OR “REGN-88” OR REGN88):ti,ab,kw |
|  | #34 | (secukinumab* OR Cosentyx OR “AIN-457” OR AIN457):ti,ab,kw |
|  | #35 | (sirukumab* OR “CNTO-136” OR CNTO136):ti,ab,kw |
|  | #36 | (tocilizumab* OR “RHPM-1” OR RHPM1 OR “RG-1569” OR RG1569 OR R-1569 OR R1569 OR “MSB-11456” OR MSB11456 OR atlizumab OR MRA OR “RO-4877533” OR RO4877533 OR Actemra OR Roactemra):ti,ab,kw |
|  | #37 | (upadacitinib* OR “ABT-494” OR ABT494 OR Rinvoq):ti,ab,kw |
|  | #38 | MeSH descriptor: [Ustekinumab] explode all trees |
|  | #39 | (ustekinumab* OR Stelara OR “CNTO-1275” OR CNTO1275):ti,ab,kw |
|  | #40 | (tofacitinib* OR tasocitinib OR Xeljanz OR “CP-690,550” OR CP690550):ti,ab,kw OR (“CP-690550”):ti,ab,kw |
|  | #41 | #12 OR #13 OR #14 OR #15 OR #16 OR #17 OR #18 OR #19 OR #20 OR #21 OR #22 OR #23 OR #24 OR #25 OR #26 OR #27 OR #28 OR #29 OR #30 OR #31 OR #32 OR #33 OR #34 OR #35 OR #36 OR #37 OR #38 OR #39 OR #40 |
