# Supplementary material for: Biologic therapies for the treatment of large vessel vasculitis: A systematic review and meta-analysis
Source: PLoS One. 2025 Mar 10;20(3):e0314566. doi: 10.1371/journal.pone.0314566 (PMC11893120; doi:10.1371/journal.pone.0314566)
Supplement: S4 Table — (DOCX) [file pone.0314566.s023.docx]

**S4 Table. Extended information of included studies regarding effects of biologic agents for the treatment of large vessel vasculitis.**

| **Study** | **Design** | **Drug** | **Comparison** | **Details of Study Population** | **Disease duration (years), mean±S.D.** | | **Prior immunosuppressant** | **Combined immunosuppressant** | **Drug Administration** | **Treatment Duration** |  |
| --- | --- | --- | --- | --- | --- | --- | --- | --- | --- | --- | --- |
|  |  |  |  |  | **Biologics** | **Comparator** |  |  |  |  |  |
| Hoffman, G. S. et al. 2007 | double-blind multicenter RCT | IFX | Placebo | newly diagnosed GCA patients in GC-induced remission | NR | NR | Yes | GC tapered according to a predefined schedule | IFX, 5 mg/kg at 0, 2, and 6 weeks and Q8W thereafter | 22 weeks in the absence of early termination |  |
| Martínez-Taboada, V. M. et al. 2007 | double-blind multicenter RCT | ETA | Placebo | biopsy-proven GCA controlled with GC therapy with side effects secondary to GC | 0.83 (0.23, 2.08)* | 0.83 (0.23, 2.08)* | Yes | GC tapered according to a predefined schedule | ETA, 25mg W2D, subcutaneously | 12 months |  |
| Seror, R. et al. 2013 | double-blind multicenter RCT | ADA | Placebo | newly diagnosed GCA patients | NR | NR | NR | GC tapered according to a predefined schedule | ADA, 40 mg at weeks 0, 2, 4, 6, 8, 10, subcutaneously | 10 weeks |  |
| Villiger, P. M. et al. 2016 | double-blind single-center RCT | TCZ | Placebo | GCA patients with humorally active at inclusion (erythrocyte sedimentation rate of ≥40 mm/h, and C-reactive protein level of ≥20 mg/L) | NR | NR | Yes | GC tapered according to a predefined schedule | TCZ, 8 mg/kg Q4W, intravenously injection | 52 weeks |  |
| Langford, C. A. et al. 2017 | double-blind multicenter RCT | ABA | Placebo | newly diagnosed or relapsing GCA patients in abatacept and GC-induced remission | 2.08±3.56 | 1.22±1.83 | Yes | GC tapered according to a predefined schedule | ABA, 10 mg/kg monthly, intravenous infusion | 12 months in the absence of early termination |  |
| Stone, J. H. et al. 2017 | double-blind multicenter RCT | TCZ | Placebo | GCA patients with active status within 6 weeks and a history of GCA-attributed elevated ESR | TCZ Q1W group, 0.84±1.55; TCZ Q2W group, 0.71±1.37 | 1.00±1.56 | Yes | GC tapered according to a predefined schedule | TCZ, 162 mg Q1W subcutaneously;TCZ 162 mg Q2W subcutaneously | 52 weeks |  |
| Schmidt, W. A. et al. 2020 | double-blind multicenter RCT | SRK | Placebo | GCA patients with active disease status at the discretion of the investigator | NR | NR | Yes | GC tapered according to a predefined schedule | SRK, 100mg Q2W, subcutaneously; SRK 50mg Q4W, subcutaneously | 12 months |  |
| Cid, M. C. et al. 2022 | double-blind multicenter RCT | Mavrilimumab | Placebo | New-onset or relapsing/refractory GCA and active disease within 6 weeks of randomization | 0.66±1.28 | 0.82±1.82 | Yes | GC tapered according to a predefined schedule | mavrilimumab 150 mg subcutaneously | 26 weeks |  |
| NCT03600805 2022 | double-blind multicenter RCT | Sarilumab | Placebo | New-onset or relapsing/refractory GCA and at least one of the symptoms of GCA within 6 weeks of baseline | NR | NR | NR | GC tapered according to a predefined schedule | Sarilumab, 150mg Q2W, subcutaneously; Sarilumab 200mg Q2W, subcutaneously | 52 weeks |  |
| NCT03765788 2023 | double-blind multicenter RCT | SCK | Placebo | new onset GCA or relapsing GCA | NR | NR | NR | GC tapered according to a predefined schedule | SCK 300mg subcutaneously at baseline, Week 1,2,3,4 and then every 4 weeks | 48 weeks |  |
| Langford, C. A. et al. 2017 | double-blind multicenter RCT | ABA | Placebo | newly diagnosed or relapsing TAK patients in ABA and GC induced remission | 6.93±5.84 | 3.52 ±4.89 | Yes, ABA and GC | GC tapered according to a predefined schedule | ABA, 10 mg/kg Q4W, intravenous infusion | 12 months after enrollment of the final patient if not meeting criteria for early termination |  |
| Nakaoka, Y. et al. 2018 | double-blind multicenter RCT | TCZ | Placebo | relapsing TAK patients in GC induced remission for at least 1 week | 6.46±7.37 | 3.57±4.03 | Yes, GC | GC tapered according to a predefined schedule | TCZ, 162 mg Q1W, subcutaneous injection | median duration, 19 weeks in the TCZ group, 12.86 weeks in the placebo group |  |
| Kong, X. et al. 2018 | prospective cohort study | TCZ | CTX | TAK patients in an active disease state | 1.70±2.77 | 0.80±1.57 | Yes | Yes. GC except for one partcipant in the TCZ group. Two partcipant in the TCZ group were also treated with leflunomide (20 mg/d) and MTX (10 mg/w). | TCZ, 8 mg/kg with the accumulated dosage of 3751.11±4483.82mg | 6 months |  |
| Pan, L. et al. 2020 | retrospective cohort study | TCZ | CTX, MTX | TAK patients with coronary artery involvement | 2.97±0.88 | 3.2±1.28 | Yes | Yes. TCZ group, 6 with MTX and 6 with GC. | TCZ, 8mg/kg Q4W, intravenously | 6 months |  |
| Kong, X. et al. 2022 | prospective cohort study | TOF | MTX | TAK patients with active disease | 3.18±4.05 | 1.69±2.79 | Partial yes | Yes. GC tapered according to a predefined schedule | TOF, 5mg bid | 12 months |  |
| Liao, H. et al. 2022 | retrospective cohort study | TCZ | CTX | TAK patients | 4.46±7.38 | 6.96±11.25 | Yes | Yes. TCZ group, 20 with MTX, 3 with MMF, 1 with HCQ, and 24 with GC. | TCZ, 8mg/kg Q4W, intravenously | 6 months |  |
| Wang, J. et al. 2022 | prospective cohort study | TOF | LEF | TAK patients with active disease | 3.09±3.73 | 0.86±1.48 | Partial yes | Yes. GC tapered according to a predefined schedule | TOF, 5mg bid | 12 months |  |
| Yoshida, S. et al. 2023 | retrospective cohort study | TCZ | MTX AZA | Patients with active TA | 5.96±9.82 | 6.33±7.97 | Yes | Yes. GC for TCZ group | TCZ, 162mg QW subcutaneous injection | median duration, 40 weeks in the TCZ group, 37 weeks in the non-TCZ group |  |

ABA = abatacept; ADA = adalimumab; CTX = cyclophosphamide; ETA = etanercept; GC = glucocorticoids; GCA = giant cell arteritis; IFX = infliximab; LEF = leflunomide; MTX = methotrexate; NR = not reported; RCT = randomized controlled trials; SCK = secukinumab; SRK = sirukumab; TAK = Takayasu’s arteritis; TCZ = tocilizumab; TOF = tofacitinib.
