# Supplementary material for: Biologic therapies for the treatment of large vessel vasculitis: A systematic review and meta-analysis
Source: PLoS One. 2025 Mar 10;20(3):e0314566. doi: 10.1371/journal.pone.0314566 (PMC11893120; doi:10.1371/journal.pone.0314566)
Supplement: S5 Table — (DOCX) [file pone.0314566.s024.docx]

**S5 Table. Definitions of the success of achieving glucocorticoid tapering in different studies.**

| **Study** | **Definition of the success of achieving GC tapering** |
| --- | --- |
| Hoffman, G. S. et al. 2007 | patients whose glucocorticosteroid dosage was tapered to 10 mg per day |
| Martínez-Taboada, V. M. et al. 2007 | patients who withdraw the corticosteroid therapy and controlling the disease activity at the end of the 12 months |
| Villiger, P. M. et al. 2016 | patients whose prednisolone dose tapered to 0 mg per day. |
| Langford, C. A. et al. 2017 | A standardized prednisone taper was applied to both treatment arms and all patients had reached a prednisone dosage of 20 mg per day at the time of randomization and had discontinued prednisone at week 28. |
| Cid, M. C. et al. 2022 | Patients were considered to have completed glucocorticoid taper if by week 26 they were receiving 1 mg/day for patients who had a starting dose of 60 mg/day, or 0 mg/day for patients who had a starting dose of less than 60 mg/day. |
| NCT03600805 2022 | participants who did not take rescue therapy from Week 12 through Week 24 and might include the use of any excess prednisone (beyond the per protocol CS tapering regimen) with a cumulative dose of <=100 mg (or equivalent), such as those employed to manage AE not related to GCA. |
| NCT03765788 2023 | Participants on Prednisolone Dose ≤ 5mg/Day at week 28. |
| Kong, X. et al. 2022 | Patients taking GCs ≤7.5 mg/day at the 12 month. |
| Wang, J. et al. 2022 | patients with persistent remission from 6 months to 12 months and achievement of GCs ≤7.5 mg/day at 12 months. |
