# Supplementary material for: Biologic therapies for the treatment of large vessel vasculitis: A systematic review and meta-analysis
Source: PLoS One. 2025 Mar 10;20(3):e0314566. doi: 10.1371/journal.pone.0314566 (PMC11893120; doi:10.1371/journal.pone.0314566)
Supplement: S6 Table — (DOCX) [file pone.0314566.s025.docx]

**S6 Table. Definitions of relapse in different TAK cohort studies.**

| **Study** | **Definition of relapse** |
| --- | --- |
| Kong, X. et al. 2022 | Relapse was denoted as reactivation of disease activity (NIH criteria ≥2 points) for patients who had achieved CR or PR. CR was defined to satisfy four criteria: (1) no new/worsened systemic symptoms, (2) no new/worsened vascular symptoms or signs, (3) erythrocyte sedimentation rate (ESR) was normal (≤40 mm/hour) and (4) GC dose ≤15 mg/day. PR was defined as 2 combined with any two of 1, 3 or 4. |
| Wang, J. et al. 2022 | “Relapse” indicated reactivation of disease (NIH criteria ≥ 2 points) after patients had achieved CR or PR. “CR” was defined based on four criteria: (i) no new symptoms or worsening of systemic symptoms; (ii) no new symptoms or worsening of vascular symptoms or signs; (iii) normal ESR (≤40 mm/h); (iv) GCs dose ≤15 mg/day (6 months) or ≤10 mg/day (12 months). “PR” was defined as meeting criterion (ii) combined with any two of (i), (iii) and (iv). |
| Yoshida, S. et al. 2023 | Relapse of TA was defined as the presence of signs of relapse, as judged by the investigator, for at least two of the following four categories according to Kerr’s criteria: systematic features, elevated ESR,  features of vascular ischaemia or inflammation, and typical angiographic features |
