# Supplementary material for: Biologic therapies for the treatment of large vessel vasculitis: A systematic review and meta-analysis
Source: PLoS One. 2025 Mar 10;20(3):e0314566. doi: 10.1371/journal.pone.0314566 (PMC11893120; doi:10.1371/journal.pone.0314566)
Supplement: S7 Table — (DOCX) [file pone.0314566.s026.docx]

**S7 Table. Definitions of remission in different TAK cohort studies.**

| **Study** | **Definition of remission** |
| --- | --- |
| Kong, X. et al. 2018 | Kerr criteria(<2) |
| Kong, X. et al. 2022 | CR was defined to satisfy four criteria: (1) no new/worsened systemic symptoms, (2) no new/worsened vascular symptoms or signs, (3) erythrocyte sedimentation rate (ESR) was normal (≤40 mm/hour) and (4) GC dose ≤15 mg/day. |
| Liao, H. et al. 2022 | An NIH score ⩽1 was defined as disease remission. |
| Wang, J. et al. 2022 | “CR” was defined based on four criteria: (i) no new symptoms or worsening of systemic symptoms; (ii) no new symptoms or worsening of vascular symptoms or signs; (iii) normal ESR (≤40 mm/h); (iv) GCs dose ≤15 mg/day (6 months) or ≤10 mg/day (12 months). |
| Yoshida, S. et al. 2023 | Remission was defined as the absence of any clinical signs or symptoms of active disease. |
