# Supplementary material for: Biologic therapies for the treatment of large vessel vasculitis: A systematic review and meta-analysis
Source: PLoS One. 2025 Mar 10;20(3):e0314566. doi: 10.1371/journal.pone.0314566 (PMC11893120; doi:10.1371/journal.pone.0314566)
Supplement: S1 File — (DOCX) [file pone.0314566.s027.docx]

**Basic Information**

**Review**

| Review Title | Biologic therapies for the treatment of large vessel vasculitis |
| --- | --- |
| Reviewer |  |
| Review Date |  |

**Study**

| Study Title |  |
| --- | --- |
| First author |  |
| Year of publication |  |
| Country of publication |  |
| Publication type |  |
| Correspondence |  |

**Eligibility of included literature**

| Is the study a randomized controlled trial |  |
| --- | --- |
| Are the participants diagnosed patients |  |
| Do the interventions meet the requirements |  |
| Are there outcomes of interest |  |
| Remarks: / (If the study is not a randomized controlled intervention document, it will be excluded, and the reason for exclusion should be noted). | |

**Characteristics of the Study Subjects**

| Characteristics of the Study Subjects |  |
| --- | --- |
| Total number of participants and the number of people in each intervention and control group | - Intervention group: - Control group:   Intervention subgroups: |
| Study design |  |
| Study Subjects | Age: (year)   - Intervention group: - Control group:   Gender：（female，n，%）   - Intervention group: - Control group:   Disease duration, month   - Intervention group: - Control group:   Prior immunosuppressant:   - Intervention group: Yes - Control group: Yes   Other basic information of the participant: e.g. elderly with diabetes  Presence of comorbidities: |
| Country/study location | e.g. United States/Seniors living in community care centers or homes |
| Intervention Measures of the Intervention Group (including duration) | Specific content, method, operational standards, personnel involved, intervention time, frequency, cycle, and whether the intervention personnel were trained, etc. |
| Intervention Measures of the Control Group (including duration) | Specific content, method, operational standards, personnel involved, intervention time, frequency, cycle, and whether the intervention personnel were trained, etc. |
| Duration of follow-up | - Intervention group: - Control group: |

**Outcomes**

|  | Intervention group: | Control group: |
| --- | --- | --- |
| Total |  |  |
| Number of exclusions |  |  |
| Number of observations |  |  |
| Number lost to follow-up |  |  |

**Outcome types**

|  | rate / proportion | time / dose /concentration |
| --- | --- | --- |
| Remission |  |  |
| Relapse |  |  |
| Glucocorticoids Tapering |  |  |
| CRP | / |  |
| ESR | / |  |

- **Remission Rate**

| Dichotomous variable group | Number of events occurred | Number of events not occurred | Total |
| --- | --- | --- | --- |
| Intervention group |  |  |  |
| Control group |  |  |  |

- **Time to Remission**

| Continuous Variables | Intervention group | | | Control group | | |
| --- | --- | --- | --- | --- | --- | --- |
| Outcomes | N | Mean | SD | N | Mean | SD |
| Time to Remission |  |  |  |  |  |  |

- **Relapse Rate**

| Dichotomous variable group | Number of events occurred | Number of events not occurred | Total |
| --- | --- | --- | --- |
| Intervention group |  |  |  |
| Control group |  |  |  |

- **Time to Relapse**

| Continuous Variables | Intervention group | | | Control group | | |
| --- | --- | --- | --- | --- | --- | --- |
| Outcomes | N | Mean | SD | N | Mean | SD |
| Time to Relapse |  |  |  |  |  |  |

- **Changes in ITAS2010**

| Continuous Variables (pre-intervention) | Intervention group | | | Control group | | |
| --- | --- | --- | --- | --- | --- | --- |
| Outcomes | N | Mean | SD | N | Mean | SD |
| ITAS2010 |  |  |  |  |  |  |
| Continuous variables (post-intervention) | Intervention group | | | Control group | | |
| Outcomes | N | Mean | SD | N | Mean | SD |
| ITAS2010 |  |  |  |  |  |  |
| Continuous Variables (WMD). | Intervention group | | | Control group | | |
| Outcomes | N | WMD | seWMD | N | WMD | seWMD |
| ITAS2010 |  |  |  |  |  |  |
| Continuous Variables (SMD). | Intervention group | | | Control group | | |
| Outcomes | N | SMD | seSMD | N | SMD | seSMD |
| ITAS2010 |  |  |  |  |  |  |

- **Changes in ITAS-A**

| Continuous Variables (pre-intervention) | Intervention group | | | Control group | | |
| --- | --- | --- | --- | --- | --- | --- |
| Outcomes | N | Mean | SD | N | Mean | SD |
| ITAS-A |  |  |  |  |  |  |
| Continuous variables (post-intervention) | Intervention group | | | Control group | | |
| Outcomes | N | Mean | SD | N | Mean | SD |
| ITAS-A |  |  |  |  |  |  |
| Continuous Variables (WMD). | Intervention group | | | Control group | | |
| Outcomes | N | WMD | seWMD | N | WMD | seWMD |
| ITAS-A |  |  |  |  |  |  |
| Continuous Variables (SMD). | Intervention group | | | Control group | | |
| Outcomes | N | SMD | seSMD | N | SMD | seSMD |
| ITAS-A |  |  |  |  |  |  |

- **Glucocorticoids Tapering Rate**

| Dichotomous variable group | Number of events occurred | Number of events not occurred | Total |
| --- | --- | --- | --- |
| Intervention group |  |  |  |
| Control group |  |  |  |

- **Glucocorticoids Tapering Dosage**

| Continuous Variables (pre-intervention) | Intervention group | | | Control group | | |
| --- | --- | --- | --- | --- | --- | --- |
| Outcomes | N | Mean | SD | N | Mean | SD |
| Glucocorticoids Dose |  |  |  |  |  |  |
| Continuous variables (post-intervention) | Intervention group | | | Control group | | |
| Outcomes | N | Mean | SD | N | Mean | SD |
| Glucocorticoids Dose |  |  |  |  |  |  |
| Continuous variables (WMD). | Intervention group | | | Control group | | |
| Outcomes | N | WMD | seWMD | N | WMD | seWMD |
| Glucocorticoids Dose |  |  |  |  |  |  |
| Continuous variables (SMD). | Intervention group | | | Control group | | |
| Outcomes | N | SMD | seSMD | N | SMD | seSMD |
| Glucocorticoids Dose |  |  |  |  |  |  |

- **Accumulated Glucocorticoids Dose (mg)**

| Continuous variables | Intervention group | | | Control group | | |
| --- | --- | --- | --- | --- | --- | --- |
| Outcomes | N | Mean | SD | N | Mean | SD |
| Cumulative Glucocorticoids Dose |  |  |  |  |  |  |

- **Changes in CRP**

| Continuous variables (pre-intervention) | Intervention group | | | Control group | | |
| --- | --- | --- | --- | --- | --- | --- |
| Outcomes | N | Mean | SD | N | Mean | SD |
| CRP |  |  |  |  |  |  |
| Continuous variables (post-intervention) | Intervention group | | | Control group | | |
| Outcomes | N | Mean | SD | N | Mean | SD |
| CRP |  |  |  |  |  |  |
| Continuous variables (Changing Value) | Intervention group | | | Control group | | |
| Outcomes | N | WMD | seWMD | N | WMD | seWMD |
| CRP |  |  |  |  |  |  |

- **Changes in ESR**

| Continuous variables (pre-intervention) | Intervention group | | | Control group | | |
| --- | --- | --- | --- | --- | --- | --- |
| Outcomes | N | Mean | SD | N | Mean | SD |
| ESR |  |  |  |  |  |  |
| Continuous variables (post-intervention) | Intervention group | | | Control group | | |
| Outcomes | N | Mean | SD | N | Mean | SD |
| ESR |  |  |  |  |  |  |
| Continuous variables (Changing Value) | Intervention group | | | Control group | | |
| Outcomes | N | WMD | seWMD | N | WMD | seWMD |
| ESR |  |  |  |  |  |  |
